# Long-term myofibroblast persistence in the capsular bag contributes to the late spontaneous in-the-bag intraocular lens dislocation

**DOI:** 10.1038/s41598-020-77207-7

**Published:** 2020-11-25

**Authors:** Jovana Bisevac, Natalia S. Anisimova, Richárd Nagymihály, Olav Kristianslund, Kirankumar Katta, Agate Noer, Ilias H. Sharafetdinov, Liv Drolsum, Morten C. Moe, Boris E. Malyugin, Goran Petrovski

**Affiliations:** 1 Center for Eye Research, Department of Ophthalmology, University of Oslo and Oslo University Hospital, Kirkeveien 166, 0450 Oslo, Norway; 2 Institute of Clinical Medicine, University of Oslo, Oslo, Norway; 3 S. Fyodorov Eye Microsurgery Federal State Institution, Moscow, Russian Federation; 4 A.I. Yevdokimov Moscow State University of Medicine and Dentistry, Moscow, Russian Federation

**Keywords:** Transdifferentiation, Translational research

## Abstract

Late spontaneous in-the-bag intraocular lens (IOL) dislocation is a complication presenting 6 months or later after cataract surgery. We aimed to characterize the cells in the lens capsules (LCs) of 18 patients with spontaneous late in-the-bag IOL dislocation. Patients' average age was 82.6 ± 1.5 years (range 72–98), and most of them had pseudoexfoliation syndrome (PEX). Cells from the LCs were positive for myofibroblast (αSMA), proliferation (Ki-67, PCNA), early lens development/lens progenitor (SOX2, PAX6), chemokine receptor (CXCR4), and transmembrane (N-cadherin) markers, while negative for epithelial (E-cadherin) marker. Moreover, the cells produced abundant fibronectin, type I and type V collagen in the nearby extracellular matrix (ECM). During ex vivo cultivation of dislocated IOL-LCs in toto, the cells proliferated and likely migrated onto the IOL’s anterior side. EdU proliferation assay confirmed the proliferation potential of the myofibroblasts (MFBs) in dislocated IOL-LCs. Primary cultured lens epithelial cells/MFBs isolated from the LC of dislocated IOLs could induce collagen matrix contraction and continuously proliferated, migrated, and induced ECM remodeling. Taken together, this indicates that long-lived MFBs of dislocated IOLs might contribute to the pathogenic mechanisms in late in-the-bag IOL dislocation.

## Introduction

Cataract surgery is the most frequently performed surgical procedure in ophthalmology. Under standard conditions, the opacified lens is emulsified with ultrasound energy, aspirated from the eye, and replaced by an artificial intraocular lens (IOL) which is usually implanted in the capsular bag^1^. Despite the high performance and safety of cataract surgery, late spontaneous IOL dislocation—a severe complication with increasing frequency^2–4^, may occur 6 months after the cataract surgery^5,6^.

The implementation of continuous curvilinear capsulorhexis in cataract surgery about 30 years ago led to decreased IOL instability and dislocation in early phases. It also increased the possibility of capsular wrinkling and late in-the-bag IOL dislocation^2,4^. The risk for late IOL dislocation increases cumulatively over the years after cataract surgery^7^.

Late in-the-bag IOL dislocation results from an interplay between progressive zonular insufficiency and pulling off the wrinkled capsular bag. Ruptured zonules are a crucial step in the development of this complication^2,4^. Lens epithelial cells (LECs) that remain after removing the anterior capsule during cataract surgery are known to undergo metaplasia and produce fibrotic extracellular matrix (ECM) extensively. The amount of remained LECs is directly proportional to capsular wrinkling progression, which may further lead to capsule contraction syndrome described in 1993^8^. Besides, zonular weakening can occur before, during, and after cataract surgery. Upon late in-the-bag IOL dislocation, weakened zonular forces fail to counteract the extended capsular phimotic force, which results in zonular rupture^2,4^.

Principally, the LECs located in the germinative zone of the lens capsule (LC), near and within the lens equator of the adult lens, can proliferate, migrate and differentiate. In contrast, LECs on the anterior capsule are considered more quiescent^9^. Immunohistochemical profiling of these cells on the anterior capsule has shown they are capable of dividing as well^10^. LECs appear to respond to injury. During cataract surgery with anterior capsulotomy performance, the remaining pool of the cells under the LC can proliferate and migrate both in vitro^11–13^ and in vivo^14,15^*.*

Conditions known to predispose to late in-the-bag IOL dislocation are pseudoexfoliation syndrome (PEX)^2,3,5–7,16–22^, trauma^2,3,6,16,18–20,22^, uveitis^2,3,6,16,22^, previous vitrectomy^2,3,5,16,19,22^, increased axial length^2,5,19,21^, retinitis pigmentosa^6,19^, connective tissue disorders^23^, time after IOL implantation^20^, long phacoemulsification time and intraoperative complications^21^. Ocular comorbidity is present in 63.8–90% of all late IOL dislocations^2,3,6,18,22^. Nevertheless, there are many reported cases with still unknown predisposing factors for this complication.

For the dislocated IOLs inside the capsular bag, it remains uncertain whether the cells extracted from the lens capsular bags are able to proliferate and induce ECM remodeling.

Experimental models with explant cultures and capsular bags with or without IOL implantation have already been used to observe the residual LECs in its original matrix and spatial organization in tissue^24^.

The current knowledge regarding the histopathology of dislocated in-the-bag IOLs is limited^2^. We hereby establish an ex vivo cultivation method for explanted dislocated IOL-capsule complexes with their LC presented in toto. This experimental model would unravel whether the cells in the capsule of dislocated IOL-capsule complexes can react upon stimuli and thus contribute as key pathomechanisms of this severe complication of cataract surgery.

## Results

### Characteristics of patients with late spontaneous in-the-bag IOL dislocation

The mean time between cataract surgery and spontaneous late in-the-bag IOL dislocation was 11.2 ± 1.6 years (range 2–25; median 11.5 years). Four patients included in this study had spontaneous dislocation 19, 20 and 25 years after the cataract surgery. The average age of the 18 patients included in the study was 82.6 ± 1.5 (range 72–98 years; median 81), women slightly more commonly affected compared to men (55.6%:44.4%). The most common predisposing factor was PEX, which was present in 15 out of 18 (83.3%) eyes. Other predisposing factors were: myopia (1 patient 5.6%), vitrectomy (2 patients, 11.1%), and glaucoma (2 patients, 11.1%). In 2 patients (11.1%), no known conditions that would increase the risk for late in-the-bag IOL dislocation could be detected, while in 4 eyes (22.2%), 2 or more predisposing factors were present (Table 1).

**Table 1:** Baseline characteristics of the patients with late in-the-bag intraocular lens dislocation.

Patient no.	Spontaneous dislocation	Age	Gender M/F	The time between cataract surgery and dislocation (years)	Predisposing factors	IOL design	Institution
1	Yes	75	M	19	PEX, Vitrectomy	3-piece	OUH
2	Yes	80	F	4	Not known	Plate haptic	OUH
3	Yes	90	F	6	PEX	1-piece	OUH
4	Yes	89	F	13	PEX	3-piece	OUH
5	Yes	79	F	10	PEX	3-piece	OUH
6	Yes	80	M	11	PEX, Myopia	3-piece	OUH
7	Yes	72	F	3	PEX	1-piece	OUH
8	Yes	84	F	9	PEX, Vitrectomy	Not known	OUH
9	Yes	82	M	15	Not known	Plate haptic	OUH
10	Yes	80	F	20	PEX	1-piece	OUH
11	Yes	89	M	2	PEX	1-piece	OUH
12	Yes	98	F	25	PEX	3-piece	OUH
13	Yes	91	F	12	PEX	1-piece	OUH
14	Yes	75	M	19	PEX	3-piece	OUH
15	Yes	83	M	4	Glaucoma	1-piece	OUH
16	Yes	81	M	13	PEX, Glaucoma	1-piece	SFEMFSI
17	Yes	81	F	3	PEX	3-piece	SFEMFSI
18	Yes	78	M	13	PEX	Not known	SFEMFSI

*M/F* male/female, *OUH* Oslo University Hospital, *SFEMFSI* S. Fyodorov Eye Microsurgery Federal State Institution.

### Cells from the spontaneously dislocated IOL-capsule complexes could proliferate and/or migrate during ex vivo cultivation

**Figure 1:**
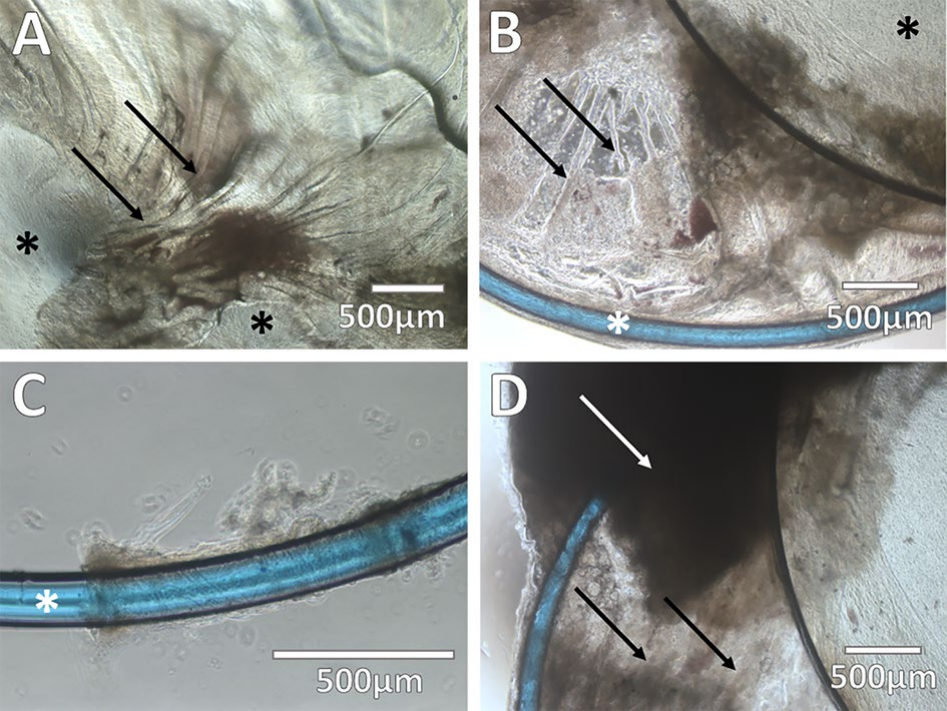
Morphological features of spontaneously dislocated intraocular lens (IOL)-capsule complexes. Direct microscopy of the dislocated IOL-capsule complexes after explantation demonstrates remodeling signs in the lens capsule. Lens capsule wrinkling and opacification with deposition of secondary cataract. (**A**) Radially aligned folds (black arrows) of the anterior capsule on the optic disc (black stars). **(B**) The appearance of fibrotic spikes (arrows) in the lens capsule between the optic disc (black star) and haptic (white star) of the IOL. (**C**) Fibrotic lens capsule tissue remaining wrapped around the haptic (white star) after a dislocation. (**D**) The appearance of Soemmering’s ring formation (white arrow) between the remaining anterior capsule and peripheral posterior capsule, as well as the opacified posterior capsule (black arrows). The scale bar is shown accordingly.

IOL-capsular bag complexes were extracted and cultured in toto with the LC bag kept intact. Direct microscopy of the extracted LCs with the IOL showed the presence of fibroblast-like cells in and out of the LC, and on the anterior side of the IOL optic disc in particular, near the edge of the anterior capsulorhexis opening (Fig. 1A,B). Cells presented in the LC showed the ability to proliferate and/or migrate ex vivo in culture conditions during a 2-week examination period. These cells could proliferate and/or migrate toward the IOL's anterior side—the IOL optic disc area, preferably (Fig. 1C,D). At the same time, proliferation and/or migration on the bottom of the culture plate was present to a lesser degree (Fig. 1E,F). Direct microscopic examination demonstrated mixed morphological features of fibroblast- and epithelial-like cells (Supplementary Fig. S1).

### Morphology of the spontaneously dislocated IOL-capsule complexes

**Figure 2:**
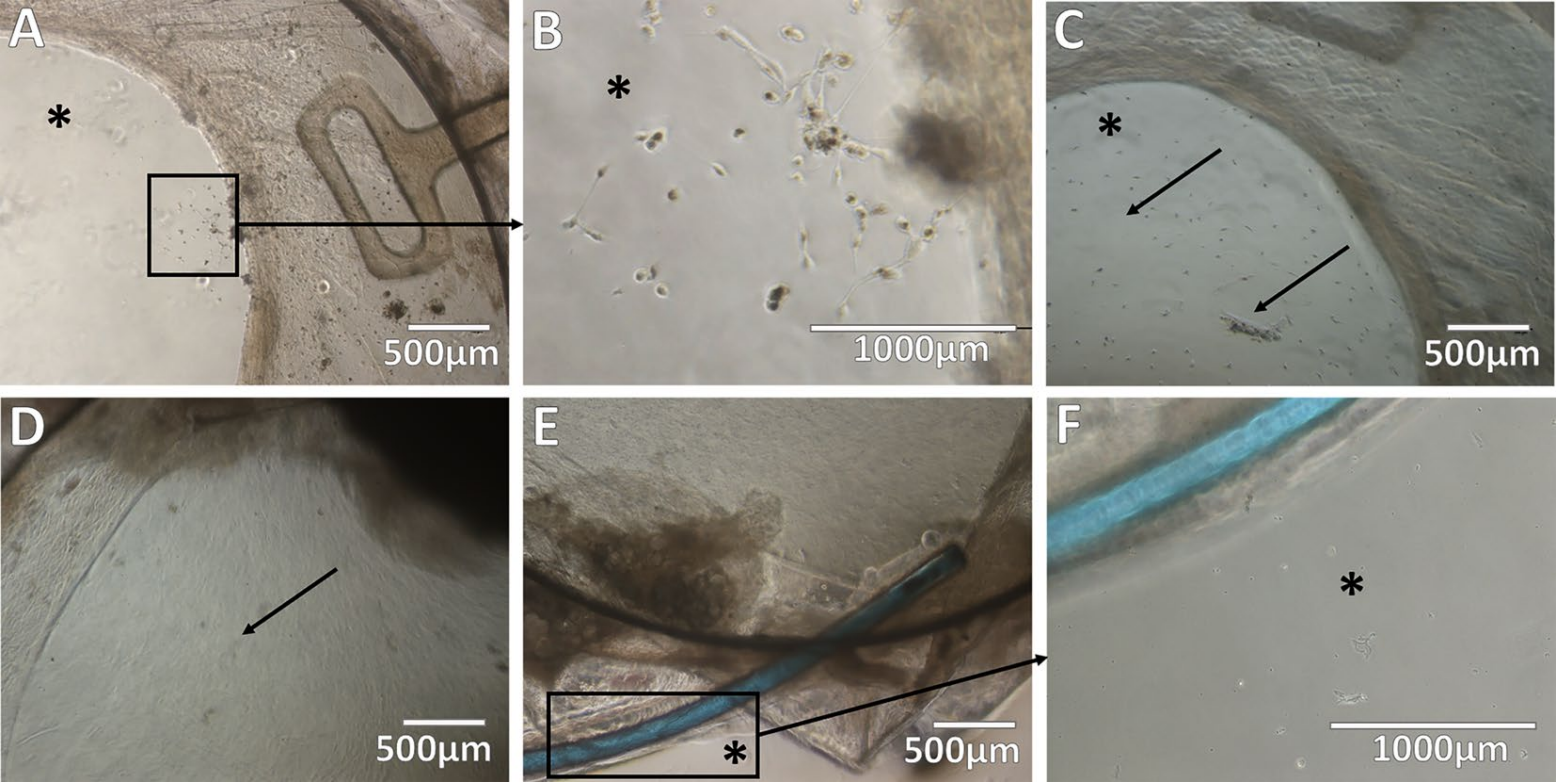
Migration and proliferation potential of the cellular content of spontaneously dislocated IOL-capsule complexes. Direct microscopy evidencing cell outgrowths capable of migrating and/or proliferating outside the lens capsule on the anterior side of the IOL material (optic disc) in ex vivo culture conditions for 2 weeks. (**A**,**B**) Anterior view of the IOL optic disc (black star) and the capsulorhexis edge of the anterior capsule (white star). Cellular outgrowth is seen on the IOL's optical surface and anterior capsule following explantation (day 0). (**C**) After 7 days of cultivation, cells (black arrows) have migrated and/or proliferated toward the center of the anterior side of the optic disc, and (**D**) by day 14 reached confluence (black arrow), completely covering the anterior side of the optic disc. (**E**,**F**) Anterior view of the peripheral edge of the dislocated IOL-capsule complex; cell migration and/or proliferation on the bottom of the culture plate (black star) outside of the dislocated IOL-capsule complex was present at a lesser degree. The scale bar is shown accordingly.

All explanted LCs contained signs of fibrosis, as well as elements of secondary cataract. Soemmering’s ring formation at the periphery, surrounding the edge of the IOL optics and haptics, with or without PCO was noted in all samples. Signs of anterior LC wrinkling in the form of radially aligned folds, as well as fibrosis of the remaining capsule surrounding IOL, were identified (Fig. 2). In only one of all explanted IOL-capsule complexes a few zonules of Zinn were present. Fibril-like material was found covering the zonules, probably being pseudoexfoliation deposits. In other explanted IOLs, zonules were absent.

### Expression of proliferation, epithelial-to-mesenchymal (EMT) and early lens development/lens progenitor genes in cells of spontaneously dislocated IOL-capsule complexes

**Figure 3:**
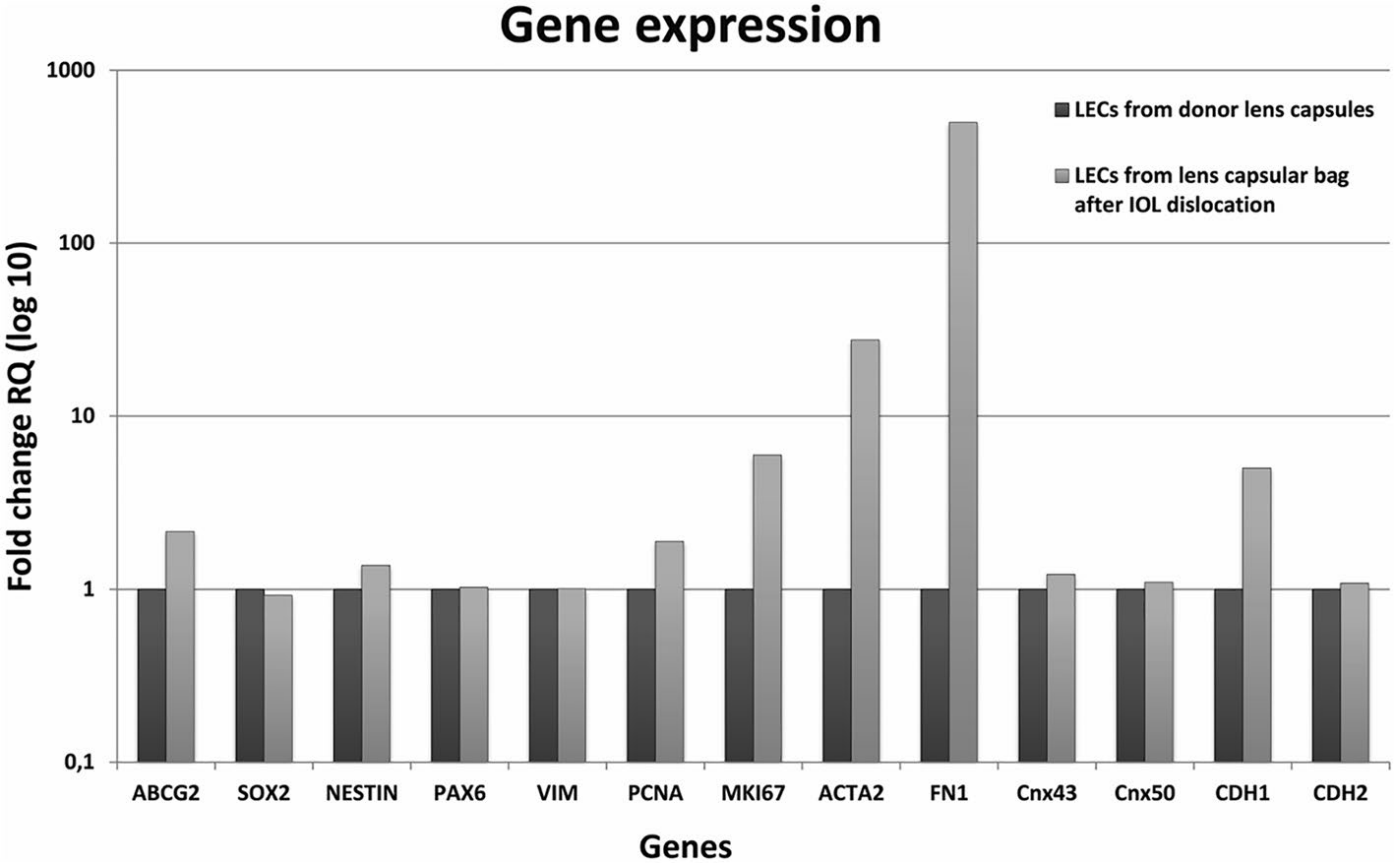
Comparative gene expression profile of lens epithelial cells (LECs)/myofibroblasts in spontaneously dislocated IOL-capsule complexes. Healthy lens capsules from cadaver donors (n = 3) were compared to LECs/myofibroblasts of lens capsules in late spontaneous in-the-bag IOL dislocation complexes (n = 3). *GAPDH* was used as a reference housekeeping gene. Genes regulating epithelial-to-mesenchymal transition (*ACTA2, FN1*) (27.5-fold and 499.8-fold, respectively) and proliferation genes (*MKI67*, *PCNA*) (6.0-fold and 1.9-fold, respectively) in LECs/myofibroblasts of dislocated IOLs were upregulated. The expression of genes regulating LEC maintenance and morphology was unchanged: *GJA1* (1.2-fold), *GJA8* (1.1-fold), *NES* (1.4-fold), and *CDH2* (1.1-fold), while it was upregulated for *CDH1* (5.0-fold) and *ABCG2* (2.2-fold) in LECs of dislocated IOLs. The fold changes in the early lens development/progenitor markers (*SOX2, PAX6*) was unchanged (0.9 and 1.0-fold, respectively), similar to that of *VIM* (1.0-fold) in both samples.

Gene expression comparison between the LECs from the LCs in dislocated IOLs and LECs from the healthy lenses using qRT-PCR showed higher expression levels of actin, alpha 2, smooth muscle, aorta gene (*ACTA2*) (27.5-fold), and fibronectin (*FN1*) (499.8-fold). In addition, gene expression of proliferation markers Ki-67 (*MKI67*) and proliferation cell nuclear antigen (*PCNA*), as well as the stem cell marker, ATP-binding cassette sub-family G2 (*ABCG2*), were also upregulated (6.0, 1.9 and 2.2-fold, respectively). The fold changes in the expression of early lens development/lens progenitor markers: sex-determining region Y-box 2 (*SOX2*) and paired box 6 (*PAX6*), as well as nestin (*NES*) gene (0.9, 1.4 and 1.0-fold, respectively), were found not to be changed, similar to the gene expression of gap junction alpha-1 and alpha-8 proteins (*GJA1* and *GJA8*) and vimentin (*VIM*) (1.2, 1.1 and 1.0-fold, respectively). Cadherin 1 (*CDH1*) showed a 5.0-fold higher expression, whereas cadherin 2 (*CDH2*) showed no expression differs from that of controls (1.1-fold) (Fig. 3).

### Myofibroblast and/or lens epithelial cell positivity for proliferation markers and early lens development/lens progenitor markers in spontaneously dislocated IOL-capsule complexes

**Figure 4:**
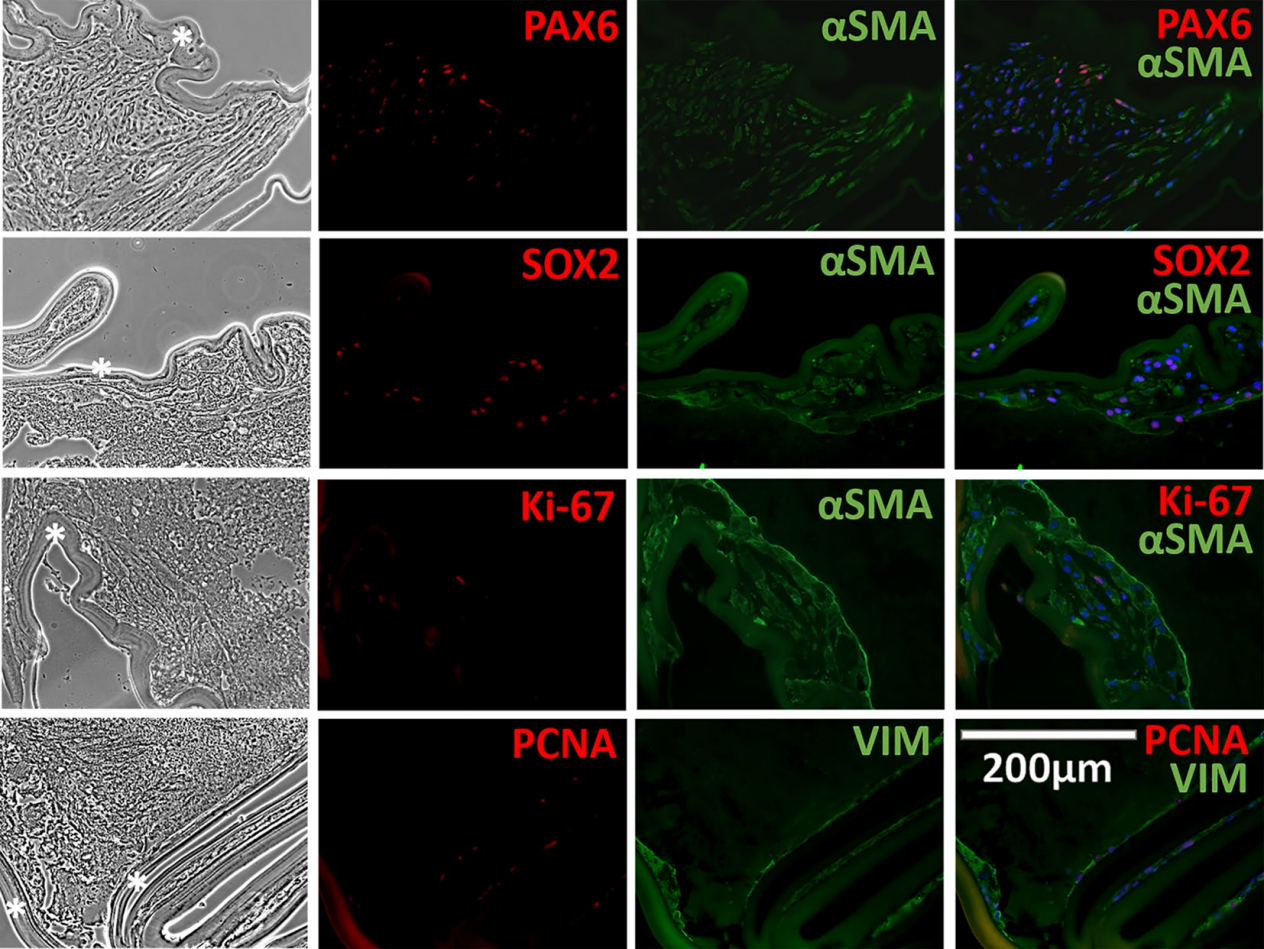
Developmental (PAX6, SOX2), proliferation (Ki-67, PCNA) and EMT (αSMA) marker analysis in late spontaneous in-the-bag dislocated IOL-lens capsule complexes. Phase-contrast (left/first column) and fluorescent immunohistochemistry (right/remaining columns) of early lens development/progenitor (PAX6, SOX2) (red), proliferation (Ki-67, PCNA) (red) markers, myofibroblast (αSMA) (green) and type III intermediate filament (Vimentin) (green) markers. Late in-the-bag spontaneously dislocated IOL-capsule complexes were cultured for 2 weeks. The majority of the lens capsule cells were myofibroblasts positioned along with the IOL material (black star). Double labeling revealed the myofibroblast (αSMA) with early lens development/progenitor (PAX6, SOX2) and proliferation (Ki-67) markers’ co-positivity, demonstrating the proliferation capacity of the individual cells. Vimentin was positive in most of the cells within the lens capsules of dislocated IOLs, with some cells also positive for PCNA. The blue color is the DAPI staining of nuclei. The scale bar is the same for all images.

**Figure 5:**
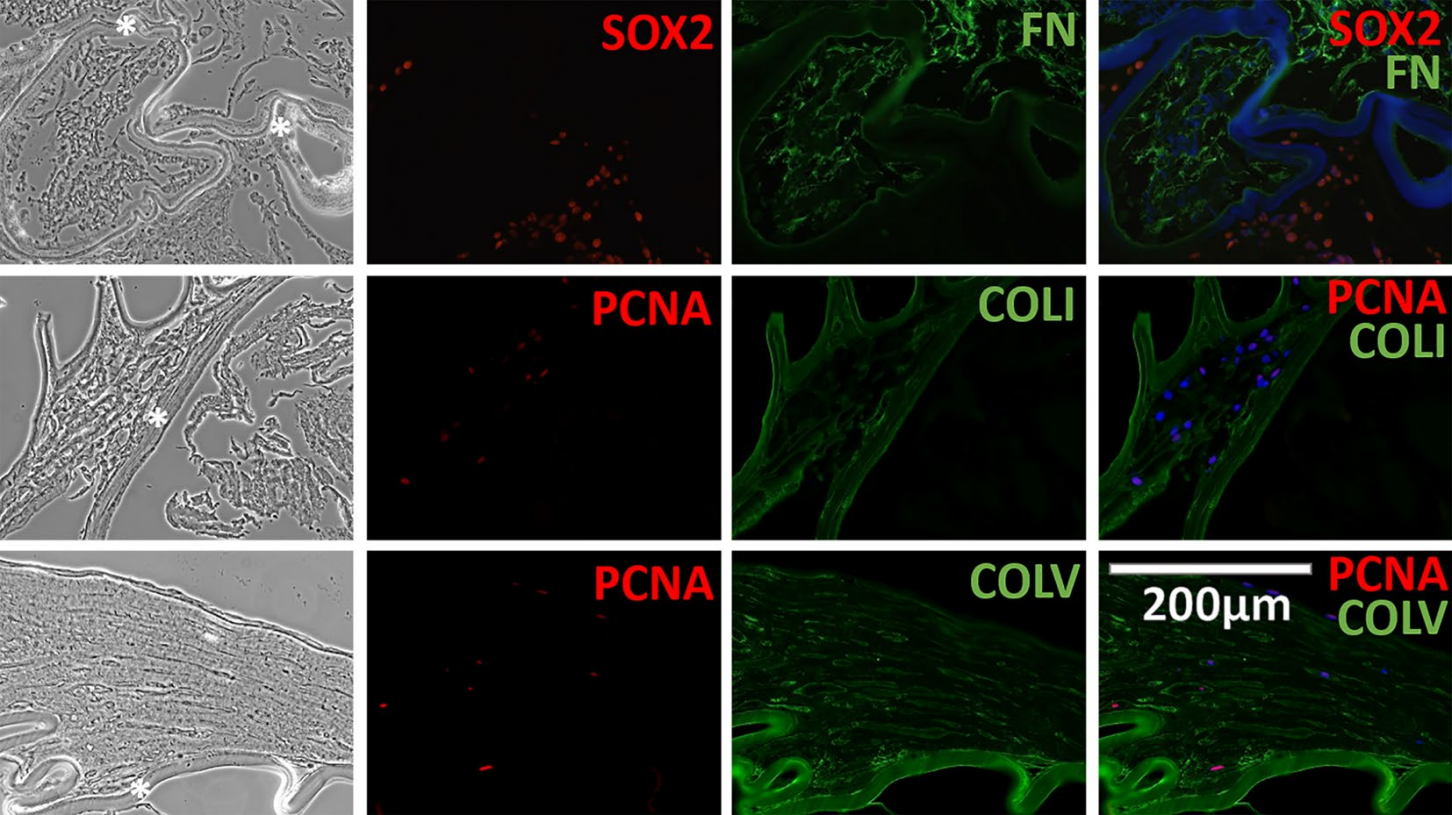
Developmental (SOX2), proliferation (PCNA) and ECM (FN, COLI, and COLV) marker analysis in late spontaneous in-the-bag dislocated IOL-capsule complexes. Phase-contrast (left/first column) and fluorescent immunohistochemistry (right/remaining columns) of fibrotic markers in the extracellular matrix (ECM) (green): fibronectin (FN), collagen I (COLI), and collagen V (COLV) production near the IOL material in dislocated IOL-capsule complexes. Neighboring cells showed positivity for proliferation, PCNA (red), and early lens development/progenitor, SOX2 (red) markers, evidencing the presence of continuous cell division. The blue color is the DAPI staining of nuclei. The scale bar is the same for all images.

Immunohistology (IHC) of the 2-week cultured and non-cultured explanted LCs with in-the-bag placed IOL showed positivity for the alpha-smooth muscle actin (αSMA), indicating the presence of myofibroblasts (MFBs) derived from LECs through EMT. Positivity for αSMA was 89.3 ± 5.3% of all cells. Most of the αSMApositive cells were lying next to the IOL material, under LC. In addition, cells demonstrated positivity for Ki-67 and PCNA proliferation markers (3.3 ± 0.7% and 46.6 ± 4.2%, respectively). The positivity for αSMA, Ki-67, and PCNA markers was not significantly different between cultured and non-cultured samples (84.6 ± 10.8 vs. 94.1 ± 1.3%, 4.0 ± 2.2 vs. 2.6 ± 0.9%, and 49.1 ± 13.7 vs. 44.1 ± 7.6%, respectively). IOL-capsule complexes also showed positivity for VIM, a type III intermediate filament naturally present in crystallin lenses, found in all LC cells close to the IOL material and the cells present in the periphery of the samples. Double staining revealed co-localization of MFB marker (αSMA) with Ki-67, SOX2, and PAX6 (Fig. 4), whereas some PCNA were double-positive for VIM, type I collagen (COLI), and type V collagen (COLV) (Figs. 4, 5). No difference in the localization and positivity of these markers was found in the cultured vs. non-cultured cells. Negative controls for Figs. 4 and 5 are provided in Supplementary Figs. S2–S10.

### Fibrous ECM and crystallin proteins deposition in spontaneously dislocated IOL-capsule complexes

**Figure 6:**
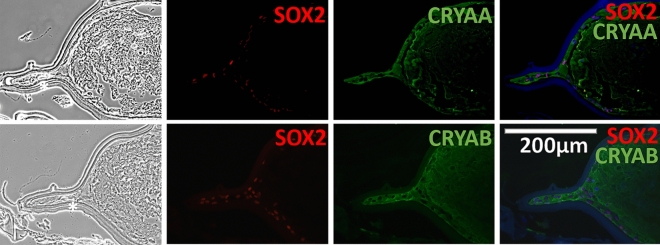
Developmental (SOX2) and lens crystallin markers analysis in late spontaneous in-the-bag dislocated IOL-capsule complexes. Phase-contrast (left/first column) and fluorescent immunohistochemistry (right/remaining columns) demonstrating the presence of crystallin proteins (green) in late spontaneous in-the-bag dislocated IOL-capsule complexes following 2 weeks of cultivation. Alpha A (CRYAA) and alpha B (CRYAB) subunits of the lens-specific protein alpha-crystallin are present in dislocated IOL-capsule complexes. Lens capsule cells next to the IOL material (black star), which were positive for SOX2 (red) and DAPI (blue), appear positive for crystallin proteins. Besides, the inner cell layers made of elongated and extended cells mostly negative for nuclear staining (DAPI), and the most inner space filled with extracellular matrix-like material, were positive for the respective markers. The scale bar is the same for all images.

Labeling for COLI, COLV, and fibronectin (FN) revealed fibrous ECM deposition. These markers were positive in all samples, mostly localized next to the cell layers surrounding the IOL. Neighboring cells showed positivity for PCNA proliferation marker and SOX2 (Fig. 5). All IOL-capsular bag histological samples contained a cell layer with positive DAPI nuclear staining lying next to the IOL material, under the LC positive for lens crystallin markers: alpha-A(α-A) crystallin (CRYAA) and alpha-B(α-B) crystallin (CRYAB). The next few layers located more into the capsular bag contained extended and elongated cells. These cells were generally negative for nuclear staining, while they also demonstrated positivity for CRYAA and CRYAB. Extracellular amorphous-like material without defined cell boundaries was filling the capsular bag’s innermost space (Fig. 6).

### EMT features in spontaneously dislocated IOL-capsule complexes

**Figure 7:**
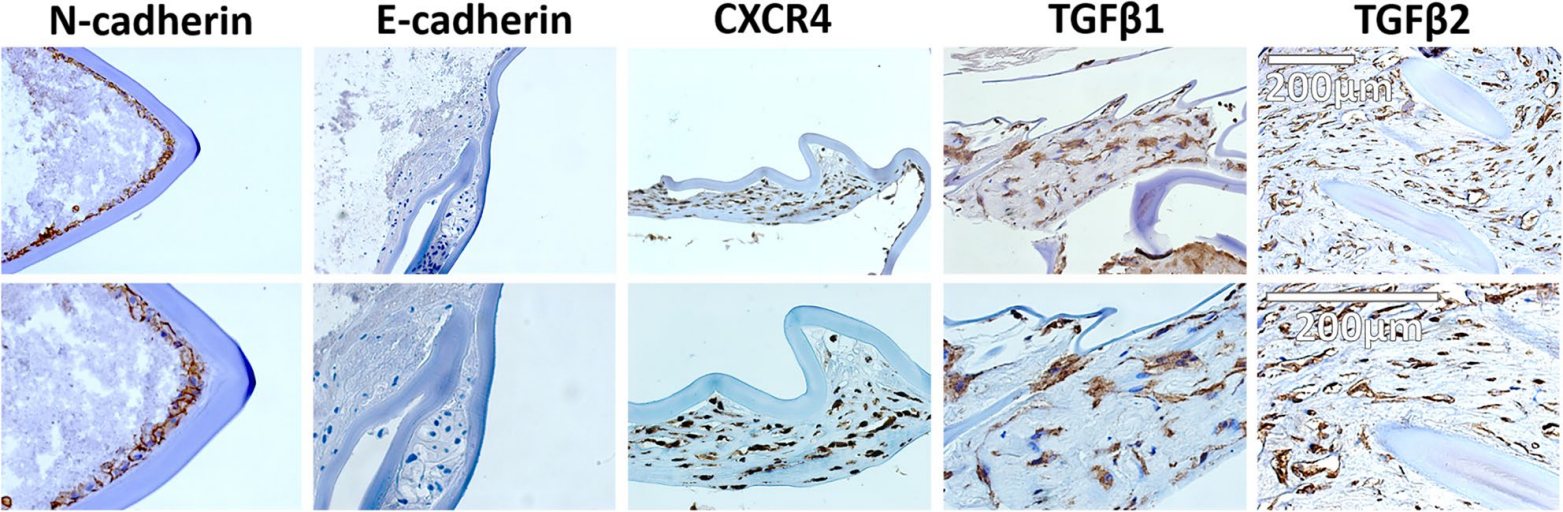
Immunohistochemical DAB staining for TGFβ1, TGFβ2, N-cadherin, E-cadherin, and CXCR4 markers in late spontaneous in-the-bag dislocated IOL complexes following ex vivo cultivation for 2 weeks. Staining revealed TGFβ1 and TGFβ2 positivity (two strong epithelial-to-mesenchymal mediators in many tissue types and lens epithelial cells) in the IOL-capsule complex tissue. Cells in the lens capsule maintained the positivity for N-cadherin, lost the positivity for E-cadherin, and transformed to myofibroblasts. This likely promoted migration of the latter, as shown by their positivity for C-X-C chemokine receptor type 4 (CXCR4), a multifunctional receptor known to play roles in migration. The scale bar is the same for all images in the same row.

E-cadherin (CDH1) staining was negative in the cells of dislocated IOL-capsule complexes. The loss of this epithelial marker in almost all cells of the lens epithelium lying next to the IOL material coincided with the presence of αSMA in them, further demonstrating their transition to MFB phenotype. The transmembrane protein, N-cadherin (CDH2), showed preserved positivity in the respective cell layer. CDH2 was also positive in the enlarged fiber-like cells of the inner cell layers without nuclei. Besides, MFB-like cells were rich in VIM, a protein naturally present in the lens. The loss of CDH1 likely promoted the cells' migration, showing positivity for the C-X-C chemokine receptor type 4 (CXCR4) known to play roles in migration. Further, labeling for transforming growth factor beta-1 (TGFβ1) and transforming growth factor beta-2 (TGFβ2), the likely major mediators of the EMT in LECs, was strong in all tissue samples (Fig. 7).

### Proliferation potential of the cells of spontaneously dislocated IOL-capsule complexes

**Figure 8:**
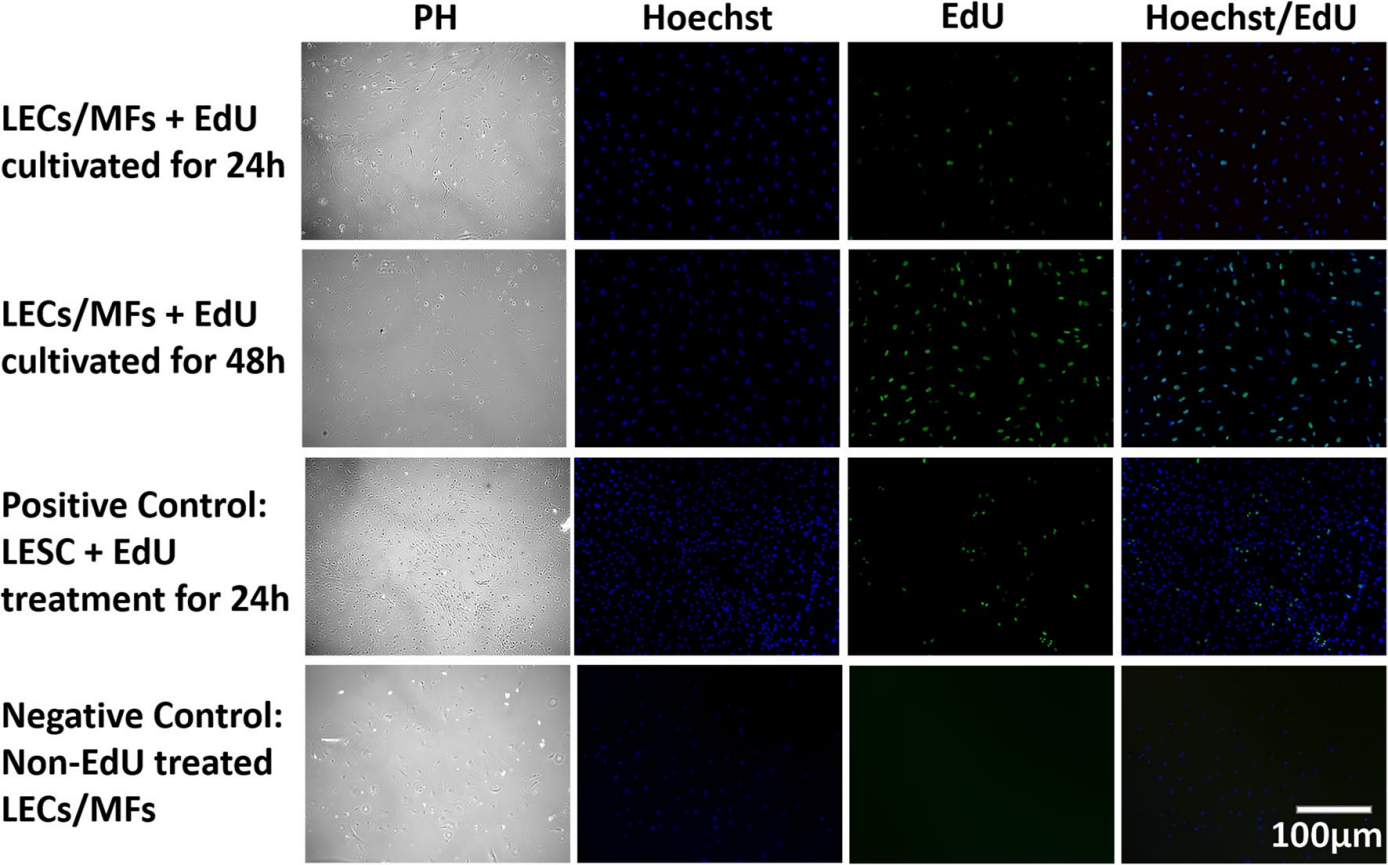
EdU cell proliferation assay on primary cultured cells of the late spontaneous in-the-bag dislocated IOL-capsule complexes. Phase contrast (PH; left/first column) and fluorescent cytochemistry (right/remaining columns) of Hoechst (blue) nuclear and EdU (green) staining are being shown. Lens epithelial cells/myofibroblasts (LECs/MFs) in the dislocated IOL-capsule complexes demonstrated the ability to proliferate after 24 h, which remained active 72 h. For comparison, proliferation of limbal epithelial stem cells (LESCs) for 24 h with EdU treatment was used as a positive control and EdU untreated LECs/MFs as a negative control. The scale bar is the same for all images.

Cultured MFB/LECs were able to divide abundantly within 3 days of cultivation, which was followed by 24 h and 72 h treatment with EdU (Fig. 8.) The proliferating ratio vs. total cells was 30.2 ± 1.4% and 68.1 ± 1.5% after 24 h and 72 h of EdU treatment.

### Primary LECs are able to induce surrounding matrix contraction

**Figure 9:**
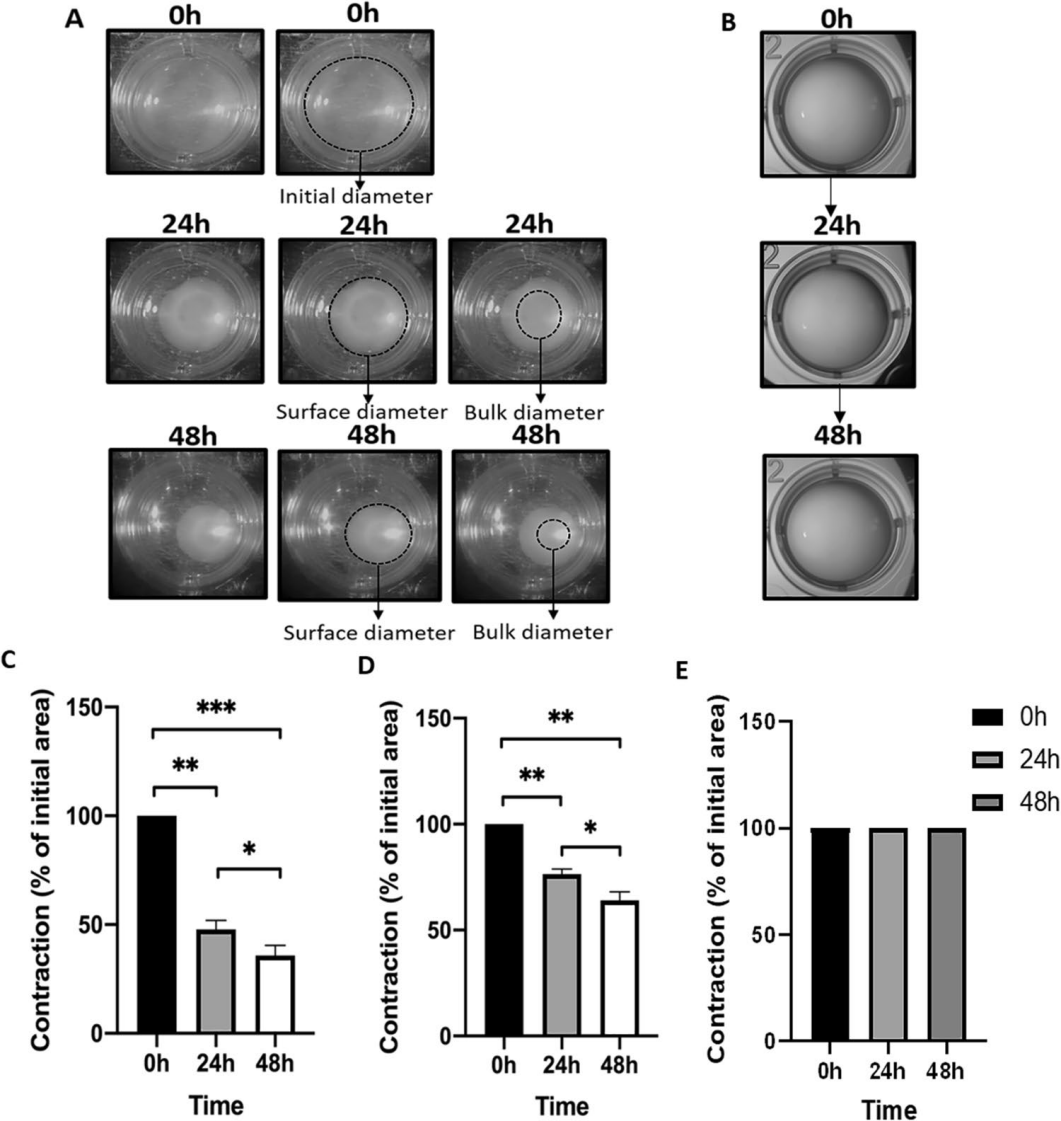
Collagen gel contraction assay on primary human LECs. (**A**) Primary LECs cultured for 4–5 weeks showed the ability to induce collagen gel contraction after 24 h and 48 h, whereas (**B**) non-cultured primary LECs could not cause such contraction of the surrounding matrix. Measurements of the representative collagen matrices (n = 4) at the initial time point and 24 h and 48 h are presented as contraction reduction of the initial area of bulk (**C**) and surface diameters (**D**) of collagen matrices of cultured and non-cultured (**E**) LECs. Bar charts present mean values and are plotted with standard error of the mean values, ***p < 0.001, **p < 0.01 and *p < 0.05.

Cultured LECs were able to contract (Fig. 9A), contrary to freshly isolated LECs from healthy lenses obtained from cadaver donors, which failed to contract collagen during the 48 h observation period (Fig. 9B). The initial collagen matrix area was reduced to 7.5 ± 0.6 and 5.6 ± 0.7 mm of bulk diameter after 24 and 48 h, respectively. In contrast, the mean values of the surface diameters after 24 and 48 h were 11.9 ± 0.4 and 10 ± 0.6 mm, respectively. The mean remodeling of collagen matrices concerning bulk diameter measurements was 52.2 ± 4.2% after 24 h and 64.1 ± 4.6% after 48 h. The contraction of the surface diameters was lower but also significant compared to the initial diameter. The collagen matrices' contraction was 23.5 ± 2.4% and 35.9 ± 3.9% after 24 and 48 h, respectively. During the first 24 h of the assay, we observed most collagen remodeling, which continued in the next 24 h to a lesser degree (Fig. 9C,D).

## Discussion

### Spontaneous late in-the-bag IOL dislocation may occur many years after cataract surgery

Late in-the-bag IOL dislocation represents a subgroup of all IOL dislocations between 6 months and 25 years after the cataract surgery^3,5,6^. It has a low incidence, as shown in a large population study with a 30-years follow-up period, with few groups reporting a more frequent occurrence lately^3,6,19,20^. A higher incidence of the condition is expected in the future due to the increased frequency of cataract surgery, as well as extended life expectancy. The risk of IOL dislocation seems to increase cumulatively over the years after cataract surgery^2,7,20^. Our study is the first to report a late in-the-bag IOL dislocation occurring 25 years after cataract surgery at the Department of Ophthalmology, Oslo University Hospital, with more than 20% of the dislocations occurring after 19 years. The mean time-period from cataract surgery to in-the-bag IOL dislocation reported here coincides with the previous reports for occurrence between 5.5 and 11.5 years^3,5,6,16–18,20,22^.

### Pseudoexfoliation is the most common underlying condition for spontaneous in-the-bag IOL dislocation

PEX is the most common risk factor for late IOL dislocation in most studies, including ours, occurring in 40–83% of the cases^2,3,5–7,16–22^. PEX patients are at significant risk of getting such dislocation^25^ even if no complication occurs before or during cataract surgery^26^. A higher histopathological positivity has confirmed the under-recording of PEX for it than what is clinically observed^27^.

PEX is present in 5–30% of adults older than 60 years globally^28^, and is more frequent in the Scandinavian populations, showing an age-dependent frequency^29^. We have previously demonstrated that the presence of PEX in patients with late in-the-bag IOL dislocation ranges between 64 and 69.8%^5,6^.

The excessive production of irregular fibrillar components in the ECM of the anterior eye chamber can contribute to glaucoma and zonular breakage and lead to phacodonesis and lens dislocations. PEX may also be associated with iris sphincter fibrosis, non-inflammatory cornea diseases such as corneal endothelial cell loss, ocular surface diseases, retinal vein occlusion^30^, and systemic manifestations such as cardio- and cerebrovascular diseases^31^.

Besides, PEX may lead to severe complications during and after cataract surgery such as PCO, intraoperative zonular rupture, higher incidence of posterior capsule defect, anterior capsular fibrosis, IOL decentration, chronic postoperative inflammation, and capsular contraction syndrome^31^.

PEX can be associated with a highly cross-linked glycoprotein-proteoglycan compound with basement membrane and elastic fibers as antigenic determinants^32^. PEX material contains mainly elastic proteins, microfibril-associated glycoprotein, latent TGFβ binding proteins, proteoglycans, matrix metalloproteinases (MMPs) and tissue inhibitor metalloproteinases, cross-linking enzymes, complement factors, and clusterin^33^. The lysyl oxidase homolog 1, which catalyzes the interstitial matrix's cross-linking, is an essential factor for developing PEX in the Scandinavian population^34^ and globally^35^ forming PEX aggregates and connective tissues modifications^36^. Inflammation, ECM remodeling, and stress conditions resemble the pathological events seen in spontaneous late IOL-dislocation^37^.

The blood-aqueous barrier in PEX eyes appears to be damaged, while the leakage of proteins into the aqueous humor increased^38^. Cataract occurrence, complications, and prolonged post-cataract rehabilitation are common in PEX patients due to an unregulated oxidative metabolism^39^.

### The lens capsule bags of spontaneously dislocated IOL-capsule complexes are wrinkled with deposition of secondary cataract masses and absent zonules

Lens capsular wrinkling can occur within 1 month after cataract surgery, and it is proportional to the amount of LECs leftover. The disbalance between the centripetal forces generated by the traction of radially oriented fibers in the LC and the centrifugal forces originating from the opposite side's zonules' traction occurs^8^. Typical radial folding and fibrotic LC morphology are noted in explanted IOL-capsule complexes. The constant remodeling of ECM, continuous proliferation and EMT of LECs are likely prevailing in centripetal forces and zonular loosening. It has been proposed that increased weight by secondary cataract masses within the capsular bag can contribute to zonular failure^3,26^. This finding is in line with our study, showing secondary cataract masses (Soemmering’s ring formation) in most of the explanted IOL-capsule complexes^3^.

Zonular fibers are typically anchored to the LC's zonular lamellae in the form of zonular fork^40^. In line with the findings of others^41^, no zonular presence was found in the IOL-capsule complexes, which could mean the weakest point of zonular stability is the anchorage to the LC. LECs within the pre-equatorial capsule seem to be changed and produce excessive PEX aggregates that may enzymatically and mechanically damage the zonular anchoring to the LC^42^. Zonules on only one explanted IOL-capsule complex in our study appeared to be reduced and covered with fibrillar-like material, probably PEX.

### Cells of spontaneously dislocated IOL-capsule complexes mostly demonstrate myofibroblast- and fiber-like cell morphology

LECs differentiate to lens fiber cells (LFCs) during lens development, enriched with tightly packed protein crystallin, allowing cells to be transparent. In pathological conditions, LECs can differentiate into immature LFCs, globularly shaped, enlarged, and filled with crystallin proteins or undergone EMT to MFBs^43^. The latter is of particular importance for cataract complications such as PCO^44,45^, anterior capsule opacification (ACO)^46,47^, or anterior subcapsular cataract (ASC) formation^43,48,49^.

LECs exposed to artificial lens material are prone to change to MFBs, which cells were positive for αSMA in our study. Patients carrying different types of IOL material and designs have the possibility of getting late in-the-bag IOL dislocation^2,3,7,27^. However, IOL types didn’t differ within in-the-bag IOL dislocation patients^20^.

Maintaining the lens capsular bag open or expanded by utilizing IOL and/or endocapsular devices (capsular tension rings) can help prevent PCO^50,51^, likely due to capsular bag expansion and inhibition of LEC migration and proliferation by the aqueous humor.

### Early lens development/progenitor markers are commonly present in the cells of dislocated IOL-capsule complexes

LECs have a defensive role in external injuries and oxidative damage, as well as regenerative potential^52^. Mouse LECs with regenerative capacity are of smaller size and localized in or anterior to the germinative zone^53,54^, as well as the anterior capsule^55^. Despite their decrease during aging, they can still be found in the adult mouse^53^.

PAX6 expression is essential in the development of all eye structures and LECs self-renewal. It is expressed mostly in the germinative zone after birth, and when co-expressed with SOX2, BMI1, and Ki-67 markers, it enables continuous division and rises to fiber cells forming lentoid in rabbits^56^. PAX6 is a robust marker maintained in all culture conditions in mouse LECs, even in TGFβ-enriched cultures^57^. We found no change in the total mRNA of *PAX6* in the cells from dislocated IOLs and protein expression in all samples analyzed.

SOX2 and SOX1/3 are mandatory transcription factors for all steps of lens development^58^. PAX6 and SOX2 activation and cooperation are obligatory for lens initiation and crystallin formation^59^. Lens progenitor cells in mice are positive for Pax6, Six3, Sox2 markers, and proliferate and move in an organized spatial manner to form the lens vesicle^60^. Besides, SOX2, PAX6, and SIX3 positive lens progenitor-like cells can derive from human embryonic cells under BMP and FGF signaling stimulus^61^. PAX6 positive cells in the germinative zone of the rabbit’s lens can proliferate, while the loss of PAX6 induces their differentiation^56^.

### Cells of spontaneously dislocated IOL-capsule complexes are continuously proliferating

SOX2, ABCG2, and Ki-67 positive LECs are more often present in LCs of younger than older patients and absent in patients above age 60 years^62^. We show LECs can proliferate under specific culture conditions in more senior living or cadaver donors. The protein expression might support an alternative theory that a sufficient amount of stimulating growth factors in older patients lacks to enhance the proliferation potential in them^12^. Indeed, LECs from elderly patients are able to proliferate enormously in response to injury^63^. In human IOL-capsule complexes models cultured immediately after performed cataract surgery, LECs could also proliferate^11^. We show proliferation in LECs/MFBs is still ongoing even years after cataract surgery through a survival mechanism kept alive by EMT. The most active cells appeared to be near the anterior capsulorhexis in culture.

Ki-67 (a marker absent in cells entering G1 phase) and PCNA (a multifunctional marker)^64^ have been used as markers of proliferation in LECs/MFBs. The difference in the gene and protein expression abundance of these two proliferation markers in our samples could be due to the diverse functional roles of PCNA or it being more specific for the LEC/MFB division.

Ki-67 and PCNA are restrictive to active replication at a specific time point; therefore, the proliferation potential was further evidenced by the EdU proliferation assay. We show MFB persistence in the capsular bag many years after cataract surgery. These long-lived MFBs possess the potential for continuous division and active, long-term remodeling of tissue, which can eventually lead to a higher cumulative risk for IOL dislocation.

### Myofibroblasts contractility in spontaneously dislocated IOL-capsule complexes may improve capsular wrinkling and tension forces on the zonules

Contractility in MFBs, fibroblasts, and mesenchymal cells correlates with their αSMA expression^65–67^. LECs/MFBs from bovine and porcine lenses, as well as from human epithelial cell-line FHL 124, could induce contraction of the surrounding matrix^68–71^. For the first time, we show the contractile nature of primary human LECs/MFBs in vitro*.*

Previous studies suggested αSMA positivity in cells to be sufficient to cause contractility by itself^65–67^. In contrast, other research indicates that TGFβ is co-required, which affects the organization of stress fibers in respective cells^13^. However, knocking down αSMA in LECs/MFBs could not prevent or decrease the matrix contraction induced by TGFβ, but enhanced it^71^.

The active treatment with TGFβ2 indeed enhanced contraction of COLI in porcine and HLEC-line FHL 124^69–71^. TGFβ2 and FGF2 had the opposite effect on collagen gel contraction, which correlated with αSMA expression levels in bovine LECs/MFBs^72^. Our LECs/MFBs derived from healthy human lenses from cadavers cultured in a medium containing 15% FCS could contract the collagen gel, which had sufficient concentration of TGFβ to induce higher expression of αSMA and redistribution of stress fibers in LECs/MFBs^73,74^.

### Long-term persistence of the myofibroblasts is of crucial importance for spontaneous late in-the-bag IOL dislocation

The EMT of LECs following cataract surgery appears to belong to one of the wound healing and tissue regeneration types. MFBs’ persistence may lead to organ fibrosis^75^. TGFβ mediates LECs transformation to MFBs ^24^, which plays an important role in the pathology of the crystallin lens and IOL-capsule complexes after cataract surgery^13,43,49,70,76^.

### TGFβ signaling may be the most accountable for long-term myofibroblast persistence and other events that lead to spontaneous late in-the-bag IOL dislocation

Higher concentrations of TGFβ in the aqueous humor exist in the patients with the two most common risk factors for late in-the-bag IOL dislocation. TGFβ1, as a major regulator of ECM, has been found in higher active- and total- concentrations in the aqueous humor of PEX patients^77^ and patients with high myopia both being at risk of getting capsular contraction syndrome^78^.

TGFβ1 and -2 seem to induce ASC with an accumulation of COLI, type III collagen (COLIII), and FN in rat and mice lenses through EMT transition of LECs. The potency of TGFβ2 is ten times higher compared to TGFβ1^49,79^. TGFβ2 could induce cell migration and wrinkling in human capsular bags resulting in PCO^70^. Positivity for respective markers in dislocated IOL-capsule complexes, concomitant with EMT hallmarks such as FN and COLI, implies their significant role in IOL dislocation pathology.

Another factor found in the EMT of LECs is a gremlin, which initiates the TGFβ/Smad2/3 signaling pathway and thus induces upregulation of α-SMA, FN, and COLI in human LECs indenpendently^80^. Besides, higher gremlin concentration is present in the aqueous humor following cataract surgery. It may promote PCO by activating Smad2/3, ERK, and AKT signaling pathways and suppressing the BMPs/Smad1/5 signaling pathway. Such changes likely led to EMT of LECs and production of fibrous proteins in ECM^81^.

Within PCO, besides MFBs, fiber-like cells have also been noted^76^. FGF in low concentrations stimulated proliferation, whereas in high concentration differentiation in the mouse LECs^57^. FGF may cause crystallin production and Soemmering’s ring formation^76^. FGF2 can also act as an antagonist of TGFβ2 in vitro, reducing the expression of αSMA while enhancing the LEC migration in combination with TGFβ2^82^. Findings show TGFβ alone can induce LECs’ transformation either to MFBs or fiber-like cells by stimulating two exclusive molecular pathways^83^. Enlarged fiber cells with seldom positivity for nuclear staining and positive for α crystallin have been found within IOL-LC complexes. The triggering factors for their formation in human LECs are not yet known.

Fiber-like cells found within the Soemmering’s ring formation and the ASC were found positive for β-crystallin^43,84^. Inhibition of TGFβ appears to be of crucial importance to maintaining the expression of αA-, αB- and β-crystallin in cultured LECs^57^. It is still unclear whether αA- and αB-crystallin within secondary cataracts and dislocated IOL complexes is initiated by TGFβ, other growth factors, or LECs’ defensive autocrine reaction.

### Transmembrane protein involvement during spontaneous late in-the-bag IOL dislocation

CDH1 is one of the most important junctional proteins between the epithelial cells, loss of which is a hallmark of EMT^85^. During normal lens development, CDH1 is conserved within LECs, which is essential for LEC morphology. Both CDH1 and CDH2 are expressed during LEC development, followed by loss of CDH1 during lens fiber cell differentiation. CDH2 is required for fiber cell development^86^. In our samples, staining for CDH1 in the epithelial cell layer next to the IOL material was negative despite the mRNA higher transcription, which is most likely due to posttranslational regulation during EMT. The negative staining of the cells of the respective layer overlapped with αSMA positivity in them. CDH2 was positive in the epithelial cell layer next to the IOL material after EMT, as well as in fiber-like cells of the more inner layers in our samples. CX43 protein is a gap junction for communication between the neighboring cells by gap junctions and a tunnel for nanotubes and extracellular vesicles^87^. Its downregulation in LECs is a sign of EMT^48^. TGFβ-induced down-regulation of genes essential for the maintenance of epithelial morphology includes *PAX6*, *GJA1*, *CRYAA*,* CDH1*, beaded filament structural protein 2(*CP49*), and zonula occludens-1 protein (*ZO-1*)^76,88^. LECs transform to MFBs in TGFβ-induced lens opacities loose expression of CDH1 and CX43^43^.

### Fibronectin may be mandatory for long-term myofibroblast persistence in spontaneous late in-the-bag IOL dislocation

FN is the major protein in ECM that binds the LECs and LC to the IOL. Fibrotic tissue under the anterior capsule remaining, posterior capsule and IOL, and Soemmering’s ring are positive for FN^89^. FN is the most recognizable marker of EMT of LECs into MFBs^83^. TGFβ2 treatment induces FN upregulation within an hour^88^. High expression of FN within dislocated IOL-capsule complexes suggests the importance of this marker in IOL spontaneous dislocation. Plasma-derived fibronectin (pdFN) upregulated integrin-mediated endogenous canonical TGFβ signaling to activate fibrotic markers such as αSMA, COLI, α5 integrin, and cellular fibronectin in embryonal chick LECs. It is involved in a feed-forward loop that prolongs fibrotic events. Also, pdFN activated LECs' differentiation to enlarged fiber-like cells similar to cells found in secondary cataract formation in response to FGF without the involvement of BMP-Smad1 signaling^90^.

FN does not seem to have a role during normal lens development and maintenance. Still, it may be mandatory for the continuous proliferation of MFBs and later fibrotic events during PCO. Mice lacking the FN gene had less expression of TGFβ and integrin signaling with sustained BMP and higher E-cadherin expression. Also, gremlin-1, the regulator of TGFβ, was decreased when compared to wild type^91^.

### Vimentin may be involved in wound-healing processes and myofibroblast movement of spontaneously dislocated IOL-capsule complexes

VIM is constitutively present in mammalian, reptilian, amphibian, and fish lenses. It is expressed in LECs and abundantly in cortical fiber cells, with a gradual decrease toward the nucleus^92^. VIM is also present in mesenchymal cells and different stages of cell development^93^. In vitro and in vivo criteria for type II EMT transition to fibroblasts include increased expression of VIM^94^, among others, and such changes have been studied in lens epithelium^95,96^. In vitro models for PCO have found LECs capable of proliferating and migrating on the IOL-LC complex's posterior capsule to be rich in VIM and actin^11^. VIM is also a central filament in wound healing of lens epithelium, expressed in cell protrusions and lamellipodial extensions of repair cells on the wound edge^97^. Our findings show no difference in the gene expression between patient and control material. VIM governs fibroblast proliferation, migration, ECM collagen deposition, and TGFβ endogenous activation that promotes EMT of keratinocytes during the wound healing process in the skin. Similar involvement may be present in MFBs of dislocated IOLs^98^.

### Collagen I and V excessive production may affect capsular bag wrinkling and robust structure of spontaneously dislocated IOL-capsule complexes

Cultured LECs can produce collagen types I, IV, V, and VI^99^. TGFβ-induced subcapsular cataract in rats showed positive staining for COLI^100^. Besides, the fibrotic capsule of opacified lenses is positive for TGFβ, αSMA, FN, COLI, and COLIII-VI types^101^. TGFβ resulted in the upregulation of αSMA, FN, pro-collagen I, and α5 integrin expression of the primary cultures^83^. Production of COLI and COLV within IOL-capsule complexes may affect capsular bags' robust structure and enhance the contraction forces.

Atypical production of COLV is found during fibrogenesis, injury repair, chronic inflammation, and cancers^102^. COLI is a known hallmark of EMT^75,94^. COLI and COLV co-propagation exists in different tissues such as corneal stroma, an interstitial matrix of smooth muscle, skeletal muscle, liver, lung, and placenta. Overexpression of COLV in obliterative bronchiolitis after lung transplantation might be due to the IL17 or TGFβ induced EMT in epithelial cells of the lung^103^. COLV, along with COLI and COLVI, has been revealed in ECM of anterior subcapsular cataract. The cells of the respective tissue were found positive for αSMA and TGFβ markers^104^. COLV likely has a more significant role in LECs pathology than it was observed formerly*.*

### Similar pathological events may be involved in posterior capsule opacification and spontaneous late in-the-bag IOL dislocation

PCO can develop a few months after cataract surgery^105^. It is present in 20–40% of eyes undergone surgery after 2–5 years^106^, and accounts for significant impairment of vision worldwide, remaining the biggest challenge in ophthalmology to resolve^105^. ND:Yag laser capsulotomy remains the only treatment for PCO, although it may cause many ocular severe complications^106^. The recurrence of Elschnig pearls can reach up to 47.6% a year after the ND:Yag laser capsulotomy^107^. During the PCO process, the LECs divide, migrate to the posterior capsule and undergo EMT or fibrous de-differentiation^45^. PCO and IOL decentration are the two most important long-standing cataract surgery complications that regularly occur together^105^. Our findings on spontaneous late in-the-bag IOL dislocation should indeed be perceived as a complementary piece in the puzzle of LECs' wound-healing response. It appears that the pathological patterns overlap in both PCO and IOL decentration and can be divided into fibrosis- and pearl-type of changes^105,106^.

Proliferation, migration, and metaplasia to MFBs of LECs leftover, followed by wrinkling and production of fibrotic components in ECM, considered the fibrosis-type. Furthermore, differentiation to atypical fiber cells producing opacified material in the form of Elschnig pearls and Soemmering’s ring formation—the pearl-type, appear to be involved in the PCO. This pattern of events is in line with our findings in dislocated IOL-capsule complexes. The wound healing response of LECs after surgery may undergo 2 phases: first-acute phase of enormous LECs proliferation and migration in the anterior capsule and equator which is dependent on the inflow of growth stimuli after surgery; second/lower/persistent phase in which autocrine mechanisms come into action^45^, and MFBs’ persist in the capsular bag, which is the most important role in spontaneous IOL dislocation. TGFβ is the major influencer in PCO, promoting EMT and suppressing the division of LECs. FGF2 inhibits TGFβ but also affects proliferation, migration, and differentiation to LFC^57,82^. Proliferation may be promoted by hepatocyte growth factor and interleukin 1 (IL-1) and interleukin 6 (IL-6) cytokines, while cell migration and contraction are likely governed by MMPs. Epithelial growth factor stimulates migration in LECs^106^.

Our findings on spontaneous in-the-bag IOL dislocation present a dynamic perpetuation of all these processes, including continuous proliferation and migration of long-lived MFBs in the capsular bag, as well as ECM remodeling leading to wrinkled, robust, and heavy capsular bag that results in zonular rupture. It is still of high priority to determine and possibly distinguish the factors involved in the complex pathophysiological steps of LEC’s response to stress conditions. The diverse strategies involved in preventing LEC migration, proliferation, and alteration to MFBs after injury may help prevent both complications.

## Conclusion

MFBs found in the LCs of dislocated IOLs continuously proliferated, migrated, and induced ECM remodeling and contraction, which over time, could cumulatively result in spontaneous IOL dislocation. Long-term MFBs’ persistence in IOL-capsule complexes may play a significant role in spontaneous dislocation.

## Methods

### Patients and biological samples

All patients’ data and tissue collections complied with the Guidelines of the Helsinki Declaration. The Regional Committees approved tissue harvesting and all laboratory procedures for Medical and Health Research Ethics, Norway (nos. 2017/2156 and 2017/418), and Local Ethical Committee, S. Fyodorov Eye Microsurgery Federal State Institution, Russian Federation (no. 81.3). Proper informed consent was obtained from each patient.

In total, 18 eyes were analyzed from 18 patients with spontaneous late in-the-bag IOL dislocation obtained after the IOL-exchange surgery (Table 1). Fifteen IOL-capsule complexes were obtained at the Department of Ophthalmology, Oslo University Hospital, Oslo, Norway from January 2018 until March 2020, and three IOL-capsule complexes the S. Fyodorov Eye Microsurgery Federal State Institution, Moscow, Russian Federation from December 2016 until March 2017. IOL-capsule complexes were collected immediately after the IOL exchange surgery and processed for further analysis.

Three IOL-capsule complexes extracted from patients (average age 80.0 ± 4.9, median 79, range 72–89 years) were snap-frozen and stored at − 80 °C for gene expression analysis; three additional ones (average age 80.7 ± 0.3, median 81, range 80–81 years) were immediately fixed in paraformaldehyde and analyzed for immunohistochemistry; and three more (average age: 83.0 ± 4.4, median 84, range 75–90 years) were proceeded to ex vivo cultivation. Three IOL-capsule complexes were immediately trypsinized and cultivated for the EdU proliferation assay (average age 83.0 ± 4.6, median 83, range 75–91 years). Tissue samples of six IOL-capsule complexes with poor quality due to the preparation process were excluded from further observations. Eleven (3 eyes + 8 eyes) LCs obtained from cadaveric donors within 24 h of biological death were used for comparison with the LCs in the late in-the-bag dislocated IOLs (3 donors, 3 eyes, average age 81.0 ± 4.9, median 80, range 73–90 years), as well as for cell culturing and contraction assays (4 donors, 8 eyes, average age 84.0 ± 3.2, median 82.5 range 78–93 years).

### Ex vivo cultivation and culture conditions

All reagents were purchased from Sigma-Aldrich (St. Louis, Mo, USA) unless otherwise stated. Extracted IOL-capsular bag complexes were immediately plated into 6-well cell culture plates (Corning^®^ Costar^®^ TC-treated multiple Well Plates) in DMEM/F-12 (31,331,028, Invitrogen, Carlsbad, CA), supplemented with 10% Fetal Calf Serum (FCS, ZQT 8811668431) and 1% penicillin–streptomycin (PS, Pen-Strep 100 U/ml, P4333) and maintained in a humidified 5% CO2, 95% air incubator at 37 °C. The LC with IOL and the outgrowing cells cultivated for 2 weeks in 2.5 ml of the medium in a 6-well plate, which was then changed every other day.

### LECs cultures and culture conditions

After removing the cornea with its limbal ring from the rest of the cadaveric eye, the lens was carefully separated from the iris, washed in Hanks’ Balanced Salt Solution (HBSS, H6648), and the LC was carefully peeled off. Two LCs from the same donor were pooled together and trypsinized in 0.25% Trypsin (T4049), thereafter blocked with DMEM/F-12 containing FCS, filtered (MACS SmartStrainer 70 μm, Miltenyibiotec) and centrifuged for 10 min on 1500 rpm at 22 °C. After cell counting, the cells were plated in the 24-well plates (Corning^®^ Costar^®^ TC-treated multiple Well Plates) in DMEM/F-12 medium supplemented with 15% FCS, 1% PS and cultured for 4–5 weeks. The medium was changed every other day.

### Real-time quantitative RT-PCR (qRT-PCR)

Total RNA was isolated from the IOL-capsular bag complexes and LCs from cadaveric donors by pooling three individual donors. RNeasy Micro Kit (Qiagen, Hilden, Germany) was used for RNA isolation. RNA was treated by RNase-free DNase treatment to digest genomic DNA. RNA was quantified using spectrophotometry (Nanodrop, Wilmington, DE). Reverse transcription (RT) was performed using High capacity cDNA Reverse Transcription Kit (Applied Biosystems, Abingdon, UK) with 400 ng total RNA per 20 μl RT reaction. The resulting cDNA was then diluted to 2 ng/μl using 180 μl RNase/DNase free water. The StepOneplus Real-Time RT-PCR (Applied Biosystems) and TaqMan Gene expression assays were used for Comparative Relative Quantification. The TaqMan Gene Expression assays included predesigned primers/probes (Applied Biosystems) (Supplementary information Table S1). Data analysis was done using the 2−ΔΔCt comparative method to achieve the fold change in gene expression and standardized to the endogenous reference gene/GAPDH values. Thermocycling conditions for RT-PCR were 95 °C for 10 min, followed by 40 cycles of 95 °C and 60 °C for 1 min. All samples were run in triplicates.

### Light microscopy

Images were acquired from the cultured IOL-capsule complexes and LECs cultures on days 0, 7, and 14, using an inverted routine microscope (Nikon TS100, Nikon, Tokyo, Japan).

### Fixation of the cultured tissue

Extracted IOL-capsule complexes that have grown in plate wells were fixed in 4% formaldehyde overnight at 4 °C and dehydrated in graded alcohol series up to 100% and xylene and after that embedded in paraffin.

### Immunohistochemistry and fluorescence

Tissue sections of 3–4 μm thickness were prepared using an automatic microtome (HM 355 s, Thermo Scientific, Massachusetts, USA) and further mounted onto histological slides. Sections were deparaffinized in xylene (10 min, 2 times) and rehydrated in graded alcohol series, 100%, 96%, 70%, and distilled water. On most of the slides, heat-induced antigen retrieval was performed in a microwave for 5 min at 900 W, then 15 min at 300 W in a citrate buffer pH 6 (C9999) or by PT module (LabVision, Fremont, CA, USA).

Slides were blocked with the 5% Bovine Serum Albumin (BSA, A9418) dissolved in Dulbecco’s Phosphate Buffered Saline (DPBS, 14190-144, Thermo Fisher Scientific) for 1 h. After that, slides were immunohistologically analyzed for markers of early lens development/lens progenitor markers SOX2 and PAX6, proliferation: Ki-67 and PCNA, MFBs: αSMA, and VIM. Also, slides were characterized for ECM markers: COLI, COLV, and FN. The samples were also tested for CRYAA and CRYAB positivity. After applying primary antibodies for 1 h, samples were washed three times with the respective wash buffer. Incubation was continued with the suitable animal type secondary antibodies for 1 h. Nuclei were stained with 4′,6-diamidino-2-phenylindole (DAPI) staining.

In addition, slides were stained for respective markers: E-cadherin, N-cadherin, CXCR4, TGFβ1, and TGFβ2 by using LabVision Autostainer360 (Lab Vision Corporation, VT) and visualized using a standard peroxidase technique (UltravisionOne HRP system). The positivity for primary antibody was recognized by a secondary antibody conjugated with peroxidase-labeled polymer with diaminobenzidine (DAB). Negative and positive controls were performed accordingly for all antibodies used. Supplementary Information Table S2 summarizes the primary and secondary antibodies. Bright-field images were taken by the ZEISS Axio Observer Z1 microscope (ZEISS, Oberkochen, Germany), while fluorescent images were taken by ZEISS Axio Imager M1 fluorescence microscope (ZEISS, Oberkochen, Germany). Each experiment was performed three times, and each sample was tested in triplicates. Image J software was used to quantify cells positive for αSMA, Ki-67, and PCNA by three independent individuals. Multiple pictures were taken of each sample, and the results were averaged as percentage mean ± standard error of the mean (SEM).

### EdU cell proliferation assay

After the IOL-exchange surgery, IOL-capsule complexes were immediately washed with Dulbecco’s Phosphate Buffered Saline (DPBS, 14190-144, Thermo Fisher Scientific). They were trypsinized in 0.25% Trypsin (T4049) for 30 min, then blocked with DMEM/F-12 containing FCS, filtered (MACS SmartStrainer 70 μm, Miltenyibiotec) and centrifuged for 10 min on 1500 rpm at 22 °C. After cell counting, the cells were plated on coverslips in 24-well plates (Corning^®^ Costar^®^ TC-treated multiple Well Plates) in DMEM/F-12 medium supplemented with 15% FCS, 1% PS. The coverslips were previously coated with poly-l-lysine (P4707) for 1 h. After 48 h, cells were treated with 10 μM EdU for 24 h and 72 h so that EdU could incorporate into the DNA during cell replication. Consequently, cultured cells were washed three times with PBS containing 3% BSA and fixed with 4% formaldehyde for 20 min, then washed again with 3%BSA in PBS and permeabilized with 0.5% Triton^®^ X-100 in PBS for 20 min. Ultimately, cells were stained with Click-iT^®^ reaction cocktail made according to the manufacturer’s instructions (Click-iT™ EdU Cell Proliferation Kit for Imaging, Alexa Fluor™ 488 dye, C10337, Invitrogen™, Thermo Fisher Scientific) for 30 min protected from light. The nuclei were stained with 5 µg/ml Hoechst 33342 solution. The coverslips with cells were mounted on slides, and fluorescent images were taken accordingly. Quantification of the EdU positive cells was carried out the same as for previously mentioned markers.

### Collagen gel contraction assay

Collagen gel contraction was performed as previously described (Barczyk et al*.* 2013). 24-well plates were coated with 2% sterile filtered BSA (BSA, A2153) in Dulbecco’s Phosphate Buffered Saline (DPBS, 14190-144, Gibco) at 37 °C for 24 h. The next day, the cells from the cell cultures were washed three times with DPBS and trypsinized for 5 min at 37 °C, blocked with medium containing FCS, and centrifuged at 1500 rpm 5 min. After that, the cell pellet was washed three times in DMEM/F-12 without serum, and the cells were counted and re-suspended until the final concentration was 1 × 10^6^ cells per 100 μl. In the meantime, the collagen solution was prepared of Dulbecco’s Modified Eagle’s Media 2X (2X DMEM, SLM-202-B), 0.2 M Hepes solution pH 8.0 (H0887) and 3.1 mg/ml Purecol Type I collagen (5005, Advanced Biomatrix) and 1% PS. The coated plates were washed with PBS two times, and the cells mixed with collagen solution (1:9) were added to wells (400 μl per well). After 90 min of polymerization at 37 °C, the polymerized gel was allowed to float by adding 400 μl of DMEM/F-12. The average size of the surface and bulk diameter was measured by the ruler at the initial time point, 24 h, and 48 h after polymerization^108^. All measurements were performed in triplicates. Results were expressed as a change of average diameters and contraction of an initial area presented as a percentage of the initial diameter at each time point.

### Statistical analyses

For patient data analysis, descriptive statistical methods were used. The mean and standard error of the mean (mean ± SEM) with median and range were used for patients’ age description, whereas the percentages (%) were used to describe the patients’ comorbidities and gender. Percentages ± SEM were used to describe positivity for Ki-67, PCNA, α-SMA, and EdU protein markers. Mean ± SEM was used to describe the average diameters at each time point in the collagen contraction assay.

Statistical analyses were performed in Prism 8 (GraphPad, USA). After the data were tested for normality and p values less than 0.05 were considered to be significant, the significant differences between the two groups were determined by using a two-tailed paired Student’s t test.

## Supplementary information

Supplementary Information.
