# Supplementary material for: Long-term myofibroblast persistence in the capsular bag contributes to the late spontaneous in-the-bag intraocular lens dislocation
Source: Sci Rep. 2020 Nov 25;10:20532. doi: 10.1038/s41598-020-77207-7 (PMC7689492; doi:10.1038/s41598-020-77207-7)

Long-term myofibroblast persistence in the capsular bag contributes to the late spontaneous in-the-bag intraocular lens dislocation

Jovana Bisevac[1,2], *Natalia S. Anisimova*[3,4], *Richárd Nagymihály*[1], *Olav Kristianslund*[1], *Kirankumar Katta*[1], *Agate Noer*[1], *Ilias Sharafetdinov*[3], *Liv Drolsum*[1,2], *Morten C. Moe*[1,2], *Boris E. Malyugin*[3,4] *and Goran Petrovski*[1,2*]

[1]Center for Eye Research, Department of Ophthalmology, Oslo University Hospital, Oslo, Norway;

[2]Institute of Clinical Medicine, University of Oslo, Oslo, Norway;

[3]S. Fyodorov Eye Microsurgery Federal State Institution, Moscow, Russian Federation;

[4]A. Yevdokimov Moscow Medical and Dental State University, Moscow, Russian Federation;

Corresponding author: Goran Petrovski, M.D., Ph.D., Dr. habil; Center for Eye Research, Department of Ophthalmology, University of Oslo and Oslo University Hospital, Kirkeveien 166, 0450 Oslo, Norway; Phone: +47 9222 6158; E-mail: goran.petrovski@medisin.uio.no

Supplementary information table S1. Assays used for gene expression in RT-qPCR

Gene name	Symbol	Taqman assay ID	Company
ATP-binding cassette sub family G2	*ABCG2*	Hs01053790_m1	Applied Biosystems™
Actin, alpha2, smooth muscle, aorta	*ACTA2*	Hs00426835_g1	Applied Biosystems™
Cadherin 1	*CDH1*	Hs01023894_m1	Applied Biosystems™
Cadherin 2	*CDH2*	Hs00983056_m1	Applied Biosystems™
Gap junction protein alpha 1	*GJA1*	Hs00748445_m1	Applied Biosystems™
Gap junction protein alpha 8	*GJA8*	Hs01102028_m1	Applied Biosystems™
Marker of proliferation Ki-67	*MKI67*	Hs04260396_g1	Applied Biosystems™
Nestin	*NES*	Hs00707120_s1	Applied Biosystems™
Paired box 6	*PAX6*	Hs01088112_m1	Applied Biosystems™
Proliferating cell nuclear antigen	*PCNA*	Hs00427214_g1	Applied Biosystems™
SRY (sex determining region Y)-box 2	*SOX2*	Hs01053049_s1	Applied Biosystems™
Vimentin	*VIM*	Hs00185584_m1	Applied Biosystems™
Fibronectin	*FN1*	Hs01549976_m1	Applied Biosystems™

Supplementary information table S2. List of primary and secondary antibodies used for immunohistochemistry experiments

Antibody	Symbol	Type	Reference	Company	Host	Dilution	Clonality
SRY-box2	SOX2	primary	ab92494	Abcam	Rabbit	1:100	Monoclonal
Paired box protein Pax-6	PAX6	Primary	60433S	Cell Signaling	Rabbit	1:100	Monoclonal
Proliferation marker Ki-67	Ki-67	primary	RM-9106-S	Thermo scientific	Rabbit	1:100	Monoclonal
Proliferation nuclear cell antigen	PCNA	primary	M0879	DakoCytomation	Mouse	1:200	Monoclonal
Alpha smooth muscle actin	αSMA	primary	ab7817	Abcam	Mouse	1:100	Monoclonal
Vimentin	VIM	primary	RM-9120-S	Thermo scientific	Rabbit	1:200	Monoclonal
Collagen I	COLI	primary	ab34710	Abcam	Rabbit	1:100	Polyclonal
Collagen V	COLV	primary	Ab7046	Abcam	Rabbit	1:100	Polyclonal
Fibronectin	FN	primary	M010	Takara Bio Inc	Mouse	1:100	Monoclonal
E-cadherin	CDH1	primary	n1620	DakoCytomation	Mouse	1:50	Monoclonal
N-cadherin	CDH2	primary	m3613	DakoCytomation	Mouse	1:100	Monoclonal
Transforming growth factor beta 1	TGFβ1	primary	ab92486	Abcam	Rabbit	1:500	Polyclonal
Transforming growth factor beta 2	TGFβ2	primary	ab36495	Abcam	Mouse	1:200	Monoclonal
Alpha A Crystallin	CRYAA	primary	ab14821	Abcam	Mouse	1:100	Monoclonal
Alpha B Crystallin	CRYAB	primary	ab13496	Abcam	Mouse	1:100	Monoclonal
C-X-C chemokine receptor type 4	CXCR4	primary	Ab124824	Abcam	Rabbit	1:100	Monoclonal
Alexa Fluor® 488 donkey anti-mouse IgG		secondary	A21202	Abcam	Mouse	1:500	Monoclonal
Alexa Fluor® 488 donkey anti-rabbit IgG		secondary	A21206	Abcam	Rabbit	1:500	Monoclonal
Cy3® goat anti-rabbit IgG		secondary	A10520	Abcam	Rabbit	1:500	Monoclonal
Cy3® goat anti-mouse IgG		secondary	A10521	Abcam	Mouse	1:500	Monoclonal

Supplementary figure S1. Cell outgrowth from a late in-the-bag dislocated IOL adhered to the culture plate. Mixed morphological features of epithelial-like and fibroblastoid-like cells can be seen. Magnification 10x (A), 20x (B).

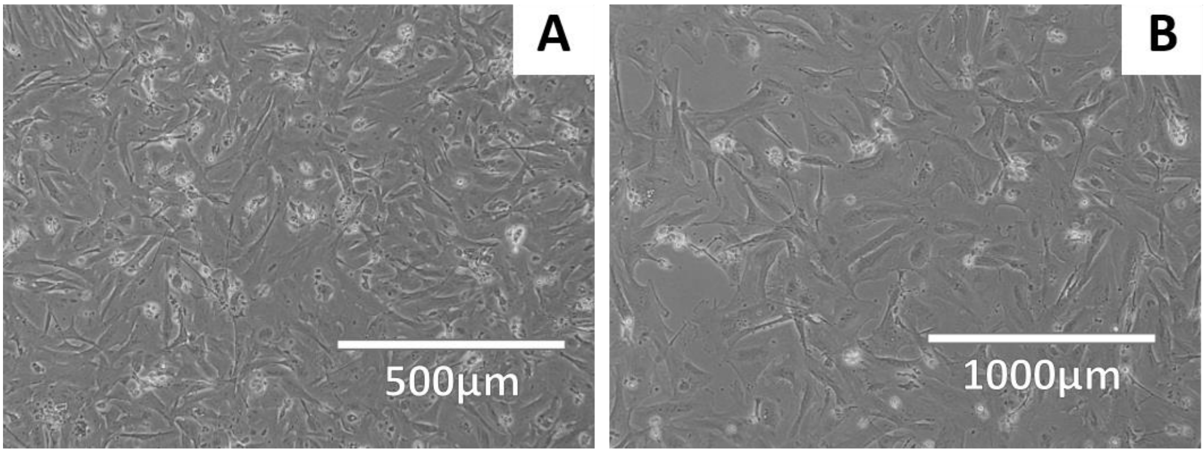

Supplementary figure S2. Phase contrast (left/first column) and fluorescent immunohistochemistry (right/remaining columns) of the late spontaneous in-the-bag dislocated IOL-capsule complexes cultured for 2 weeks**:** PAX6 (red) αSMA (green) positivity (first row) and negative control (second row). The blue color represents the DAPI staining of the nuclei. Magnification: 40x.

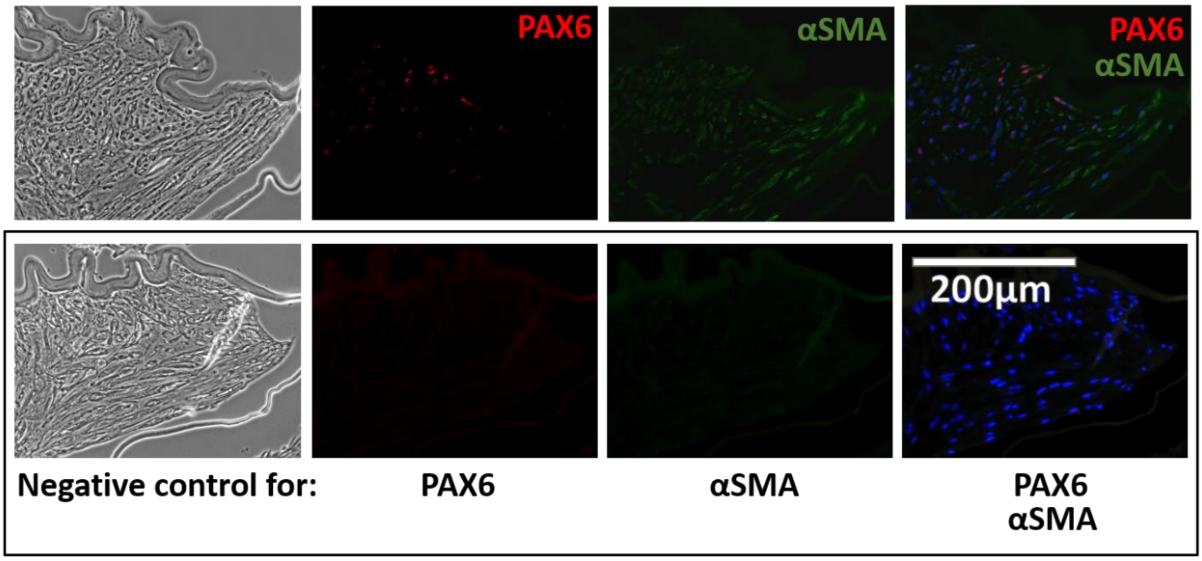

Supplementary figure S3. Phase contrast (left/first column) and fluorescent immunohistochemistry (right/remaining columns) of the late spontaneous in-the-bag dislocated IOL-capsule complexes cultured for 2 weeks**:** SOX2 (red) and αSMA (green) positivity (first row) and negative control (second row). The blue color represents the DAPI staining of the nuclei. Magnification: 40x.

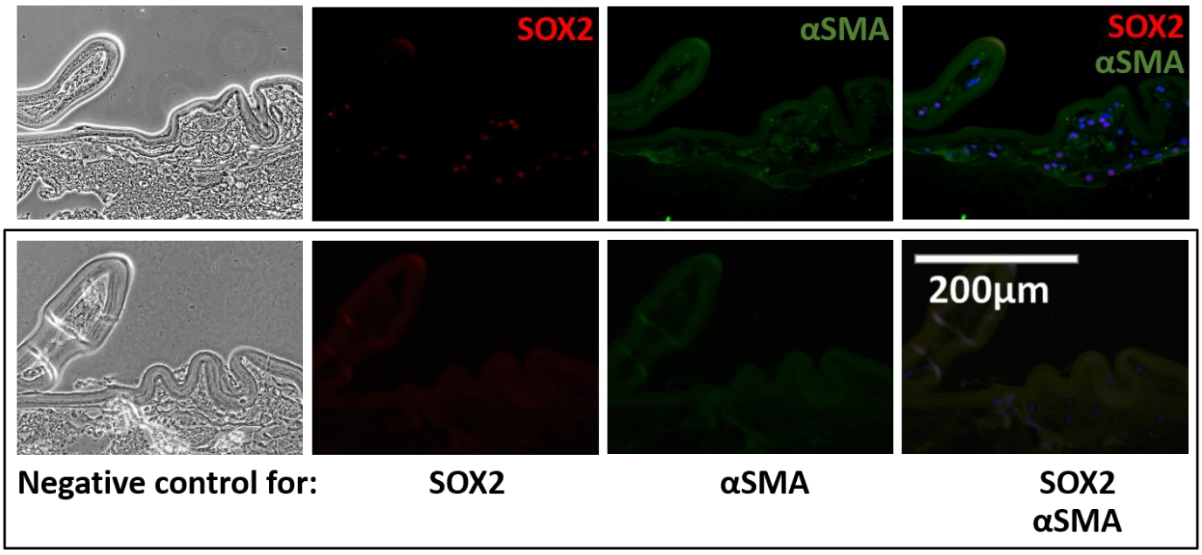

Supplementary figure S4. Phase contrast (left/first column) and fluorescent immunohistochemistry (right/remaining columns) of the late spontaneous in-the-bag dislocated IOL-capsule complexes cultured for 2 weeks**:** Ki-67 (red) and αSMA (green) positivity (first row) and negative control (second row). The blue color represents the DAPI staining of the nuclei. Magnification: 40x.

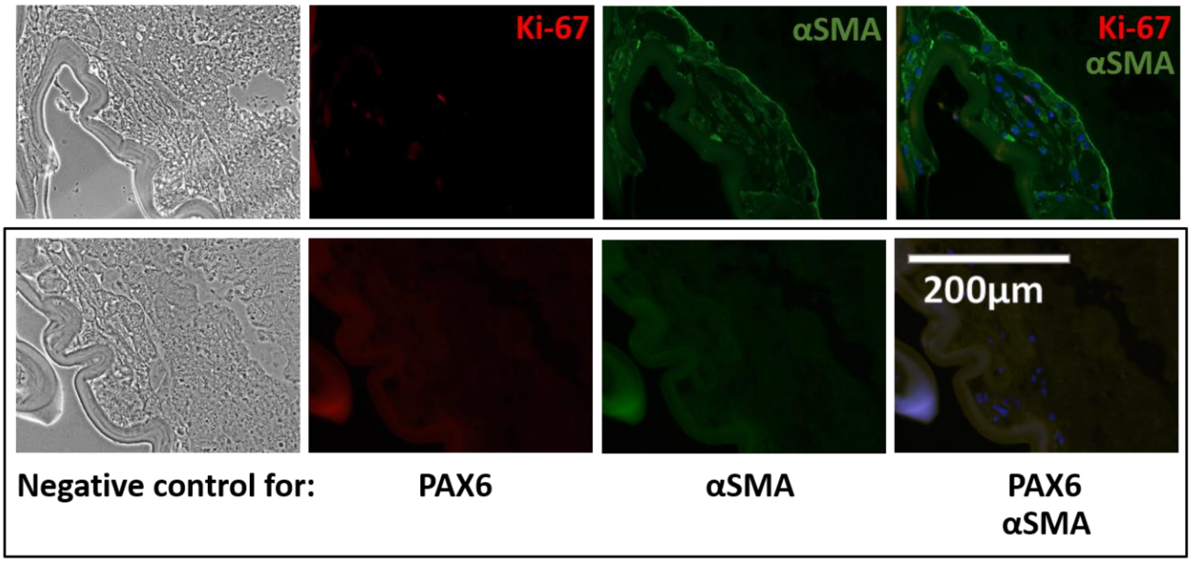

Supplementary figure S5. Phase contrast (left/first column) and fluorescent immunohistochemistry (right/remaining columns) of the late spontaneous in-the-bag dislocated IOL- capsule complexes cultured for 2 weeks**:** PCNA (red) and VIM (green) positivity (first row) and negative control (second row). The blue color represents the DAPI staining of the nuclei. Magnification 40x.

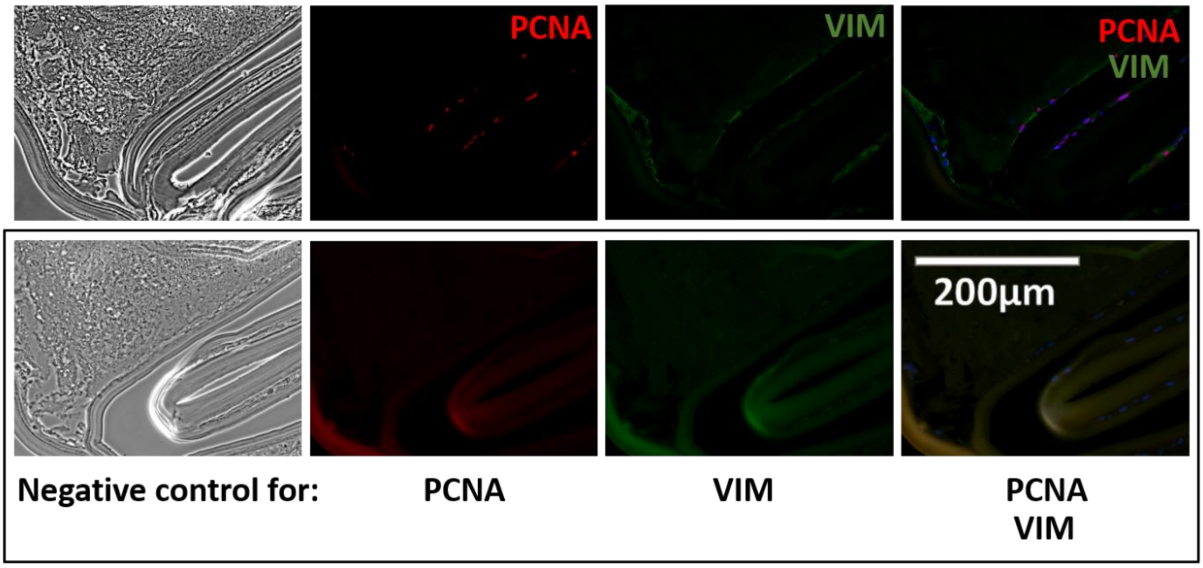

Supplementary figure S6. Phase contrast (left/first column) and fluorescent immunohistochemistry (right/remaining columns) of the late spontaneous in-the-bag dislocated IOL-capsule complexes cultured for 2 weeks: SOX2 (red) and FN (green) positivity (first row) and negative control (second row). The blue color represents the DAPI staining of the nuclei. Magnification 40x.

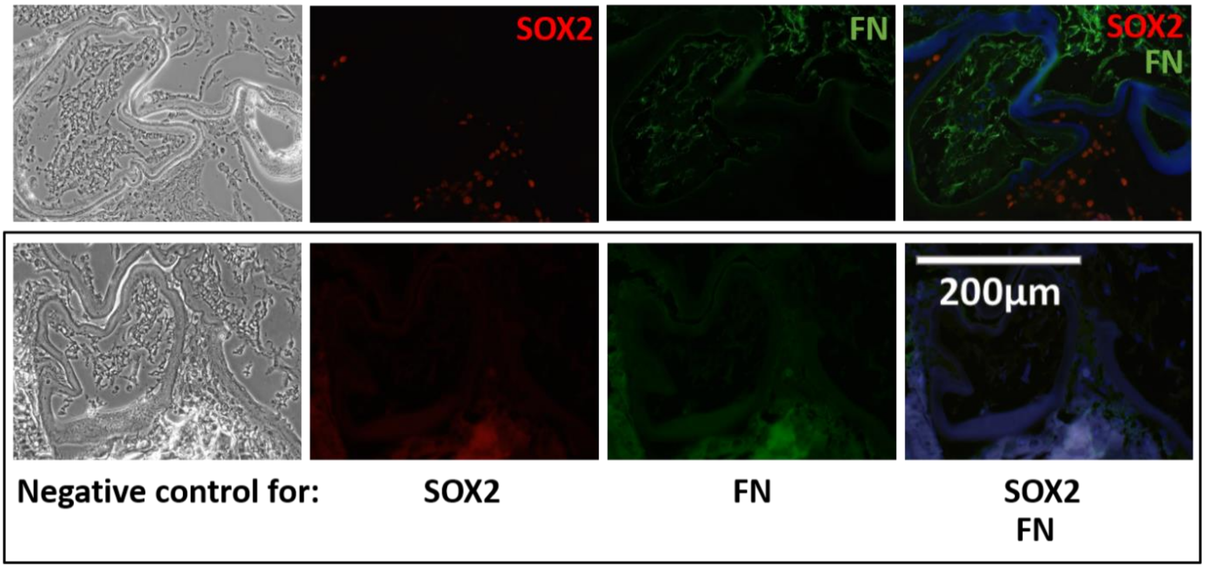

Supplementary figure S7. Phase contrast (left/first column) and fluorescent immunohistochemistry (right/remaining columns) of the late spontaneous in-the-bag dislocated IOL-capsule complexes cultured for 2 weeks: PCNA (red) COLI (green) positivity (first row) and negative control (second row). The blue color represents the DAPI staining of the nuclei. Magnification 40x.

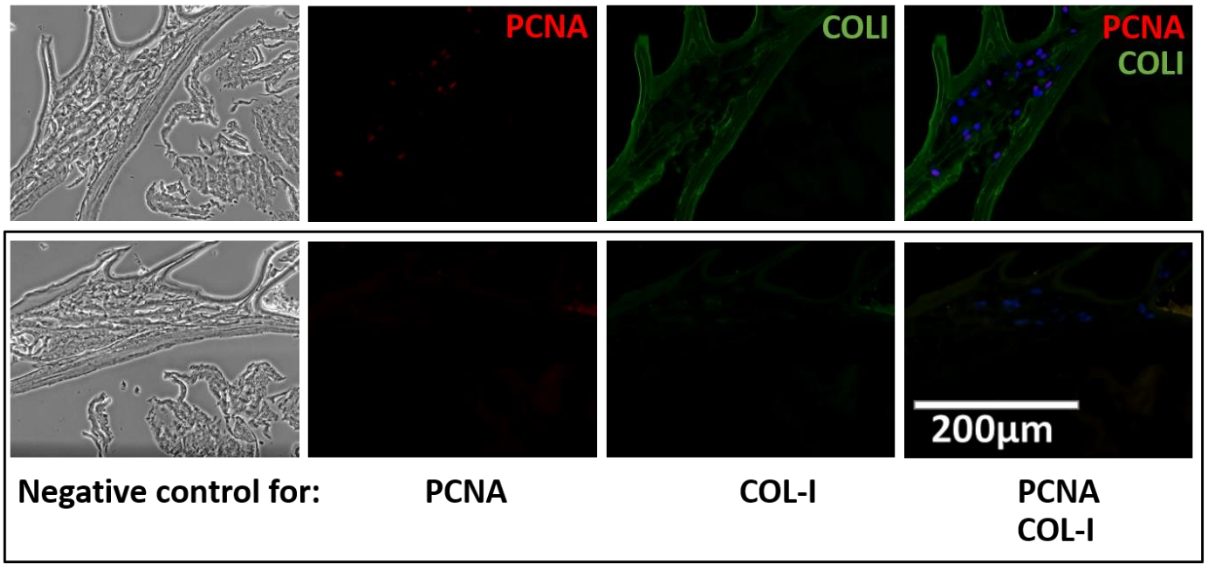

Supplementary figure S8. Phase contrast (left/first column) and fluorescent immunohistochemistry (right/remaining columns) of the late spontaneous in-the-bag dislocated IOL-capsule complexes cultured for 2 weeks: PCNA (red) COLV (green) positivity (first row) and negative control (second row). The blue color represents the DAPI staining of the nuclei. Magnification 40x.

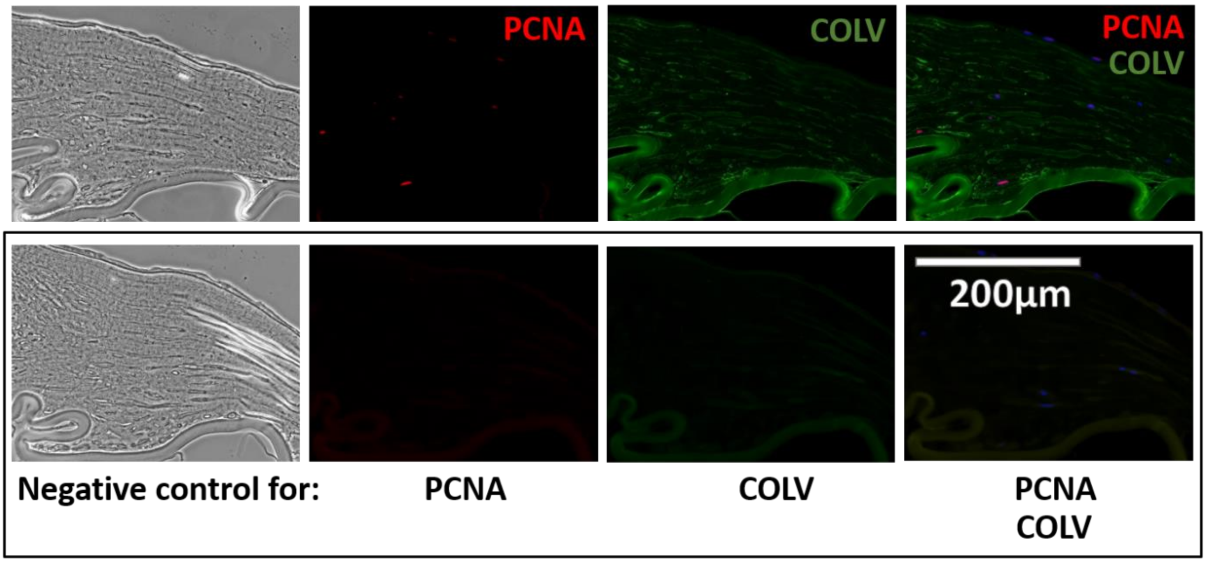

Supplementary figure S9. Phase contrast (left/first column) and fluorescent immunohistochemistry (right/remaining columns) of the late spontaneous in-the-bag dislocated IOL-capsule complexes cultured for 2 weeks**:** SOX2 (red) and CRYAA (green) positivity (first row) and negative control (second row). The blue color represents the DAPI staining of the nuclei. Magnification 40x.

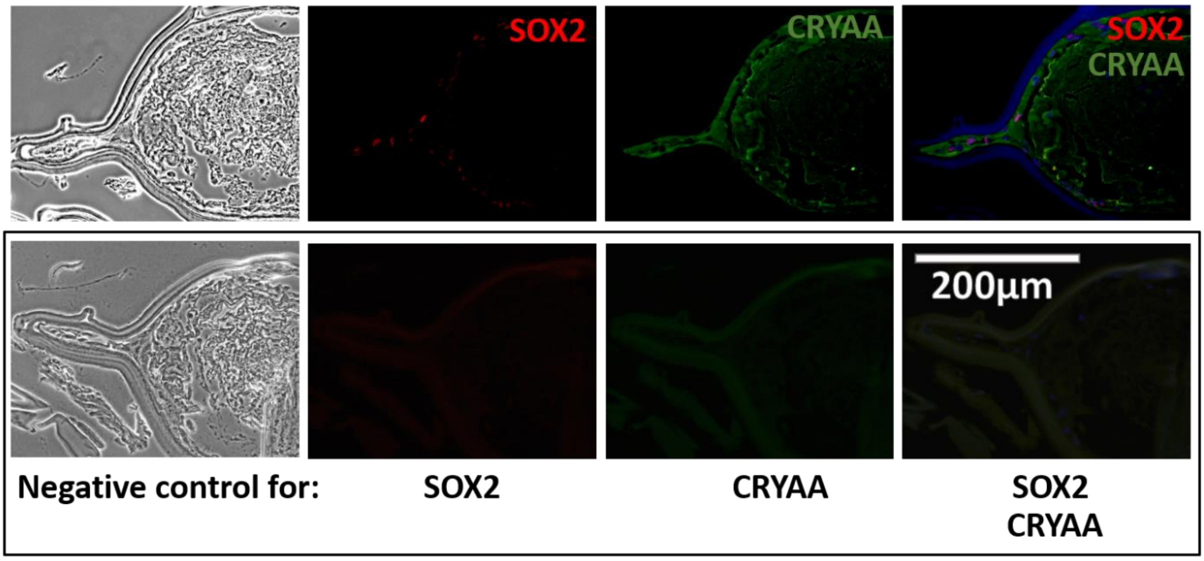

Supplementary figure S10. Phase contrast (left/first column) and fluorescent immunohistochemistry (right/remaining columns) of the late spontaneous in-the-bag dislocated IOL-capsule complexes cultured for 2 weeks**:** SOX2 (red) and CRYAB (green) positivity (first row) and negative control (second row). The blue color represents the DAPI staining of the nuclei. Magnification 40x.

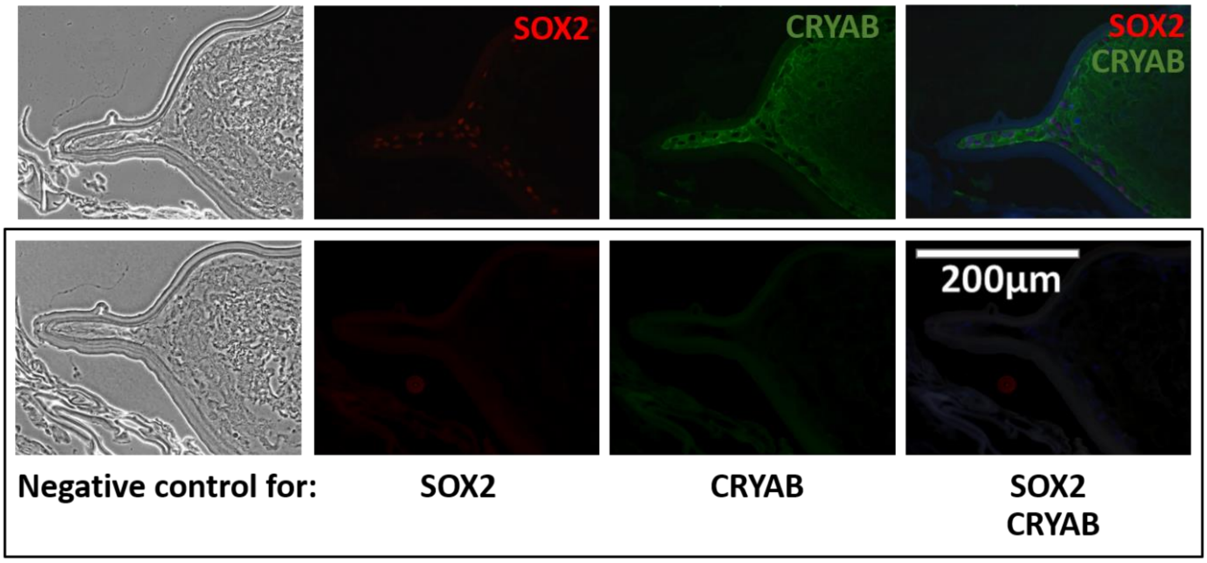

Supplement: Supplementary file 1 — Supplementary Information. [file 41598_2020_77207_MOESM1_ESM.pdf]
